# Second harmonic generation microscopy: a powerful tool for bio-imaging

**DOI:** 10.1007/s12551-022-01041-6

**Published:** 2023-01-19

**Authors:** Arash Aghigh, Stéphane Bancelin, Maxime Rivard, Maxime Pinsard, Heide Ibrahim, François Légaré

**Affiliations:** 1 Centre Énergie Matériaux Télécommunications, Institut National de La Recherche Scientifique, Varennes, QC Canada; 2 Univ. Bordeaux, CNRS, IINS, UMR 5297, 33000 Bordeaux, France; 3 National Research Council Canada, Boucherville, QC Canada; 4 Institut National de Recherche en Sciences Et Technologies Pour L’environnement Et L’agriculture, Paris, France

**Keywords:** Non-linear microscopy, SHG, Neuroimaging, Interferometry, Polarimetry

## Abstract

Second harmonic generation (SHG) microscopy is an important optical imaging technique in a variety of applications. This article describes the history and physical principles of SHG microscopy and its more advanced variants, as well as their strengths and weaknesses in biomedical applications. It also provides an overview of SHG and advanced SHG imaging in neuroscience and microtubule imaging and how these methods can aid in understanding microtubule formation, structuration, and involvement in neuronal function. Finally, we offer a perspective on the future of these methods and how technological advancements can help make SHG microscopy a more widely adopted imaging technique.

## Introduction

Despite being tremendously powerful tools, conventional linear optical microscopes suffer from scattering and a lack of optical sectioning in thick and complex samples (Helmchen and Denk 2005). Over the past two decades, second harmonic generation (SHG) microscopy has become a key method for optical imaging with many applications in materials and biomedical science. Advancements in the development of reliable and robust ultrafast mode–locked laser technologies have been pivotal for the improvement of non-linear optical microscopy techniques (Lamb 1964; Smith et al. 1974; Millard et al. 2003), especially for biomedical imaging. Using these laser sources, turn-key microscopes have been developed and are now widely spread within research laboratories.

SHG microscopy imposes a requirement: the structure of interest needs to be non-centrosymmetric (Boyd 2019), which makes it highly sensitive to filamentous proteins in biological samples (Mohler et al. 2003; Cox 2011). Otherwise, samples must be stained with appropriate SHG dyes (Campagnola et al. 2001). While this requirement limits SHG application to only a few structures, it is also a key strength since the signals are highly specific and offer sharp contrast images. Beyond that, SHG microscopy has several advantages over fluorescence imaging: it is based on an endogenous contrast (i.e., the contrast arises from the sample itself and not in a dye or fluorophore). Lastly, unlike fluorescence, SHG is free from photobleaching (the signal generated is not limited in time) and occurs instantaneously (no limitation on the laser repetition rate) (Hoover and Squier 2013).

In this review, we will first provide an overview of SHG microscopy of highly organized biological structures from its history and theoretical principles to its application to various tissues. We will then focus on several advanced SHG modalities and lastly, we will discuss SHG application in neuroscience.

### Second harmonic generation microscopy for biomedical imaging

In this section, we will briefly describe the history behind SHG microscopy, and we will provide a brief introduction of the principles behind the SHG signal generation and how it can be applied to biomedical research.

#### History

An exhaustive historical overview on SHG would start in the nineteenth century, during which Lord Rayleigh introduced the non-linearity of acoustic waves in his theory of sound (Strutt 2011; Bloembergen 1992). In this review, we will focus on the use of laser driven SHG processes to provide imaging contrast in biological samples in parallel to the development of advanced microscopy techniques. For a more comprehensive and in-depth look into the history and development of SHG microscopy, we refer to Masters and So (Masters and So 2008).

In 1931, two-photon absorption was theoretically predicted by Goeppert-Mayer (Göppert-Mayer 1931). Three decades later, in 1960, the ruby laser was created by Maiman (Maiman 1960) based on the theoretical foundation developed by Schawlow and Townes (Schawlow and Townes 1958). For more details on laser invention and its fundamental impact in science and technology, we suggest the excellent review by Siegman (Siegman 1986). Almost immediately after this discovery, different non-linear optical processes were observed starting with SHG in 1961, when Franken et al. observed frequency doubling of a ruby laser in a quartz crystal (Franken et al. 1961). At this time, the measured SHG signal was so dim that it was famously mistaken by the *Physical Review* editor as a speck of dust. In 1962, Bloembergen and Pershan derived the SHG equations and described key principles ruling light-matter non-linear interaction through an in-depth review of Maxwell’s equations (Bloembergen and Pershan 1962). For a comprehensive and detailed explanation of the fundamentals and formulations of non-linear optics, we strongly recommend the nonlinear optics book (Boyd 2019). As for biological samples, the first attempts to understand piezoelectric and pyroelectric effects in bone and tendon were realized in 1964 by Fukada and Yasuda (Fukada and Yasuda 1964) and in 1966 by Lang (Lang 1966). They demonstrated that tendon has a macroscopic polar structure using piezoelectric (Fukada and Yasuda 1964) and pyroelectric measurements (Lang 1966), although without successfully identifying the structural origin of piezoelectric and pyroelectric effects.

In parallel, the confocal microscope, originally developed by Minsky in 1955 to image unstained neural networks of the brain (Schuldt 2009Fellers and Davidson), encountered a tremendous success, leading to the first implementation of laser scanning confocal microscopy in the late 1960s. In 1974, Hellwarth and Christensen already combined SHG with optical microscopy by applying a focused laser on potassium deuterium hydrogen phosphate (KDP) crystals (Hellwarth and Christensen 1974). However, this method was solely based on very strong SHG converters as the entire field was illuminated with a CW laser. In 1977, Sheppard et al. imaged quartz with a scanning SHG microscope using a tight focusing that allowed to detect the non-linear optical signal (Sheppard et al. 1977). Simultaneously, Parry and Craig showed, using electron microscopy (EM), that collagen fibrils composing tissues, such as tendons, possess an architecture with mixed polarity with neighboring fibrils pointing in opposite directions (Parry and Craig 1977). This was later confirmed using the combination of atomic and piezoelectric force microscopy (Minary-Jolandan and Yu 2009; Harnagea et al. 2010). In 1978, Roth and Freund reported on comparative measurements between the SHG signal of a reference quartz sample and a rat-tail tendon. They found that the SHG signal was 3–4 orders of magnitude lower in the biological sample than in the reference material and already highlighted that SHG measurements could be advantageously used in vivo (Roth and Freund 1979). Finally, in 1986, Freund and Deutsch were the first to perform SHG microscopy of biological samples and proved that the macroscopic polar structure in the tendon arises from the network of fine structures that happen to be collagen fibrils, within the whole tissue volume (Freund and Deutsch 1986). In that pioneering publication, the viability of using SHG microscopy for biomedical imaging was demonstrated.

In 1990, Denk et al. introduced two-photon excitation fluorescence (2PEF) laser scanning microscopy using pulsed lasers and a modified confocal microscope (Denk et al. 1990). Following the success of 2PEF, in 1996, three-photon excitation microscopy was demonstrated (Hell et al. 1996). Although the SHG modality is older than 2PEF microscopy (Denk et al. 1990), it was forgotten for over a decade and rediscovered in 1998 (Gauderon et al. 1998; Bianchini and Diaspro 2013) and combined with 2PEF in the early 2000s in many studies (Cox et al. 2003; Hemmer et al. 2016; James and Campagnola 2021). Since then and following the progress in commercially available mode-locked lasers and user-friendly multiphoton microscopes (Zipfel et al. 2003), SHG has become a powerful method for multimodal high spatial resolution optical imaging.

#### SHG microscopy

**Fig. 1:**
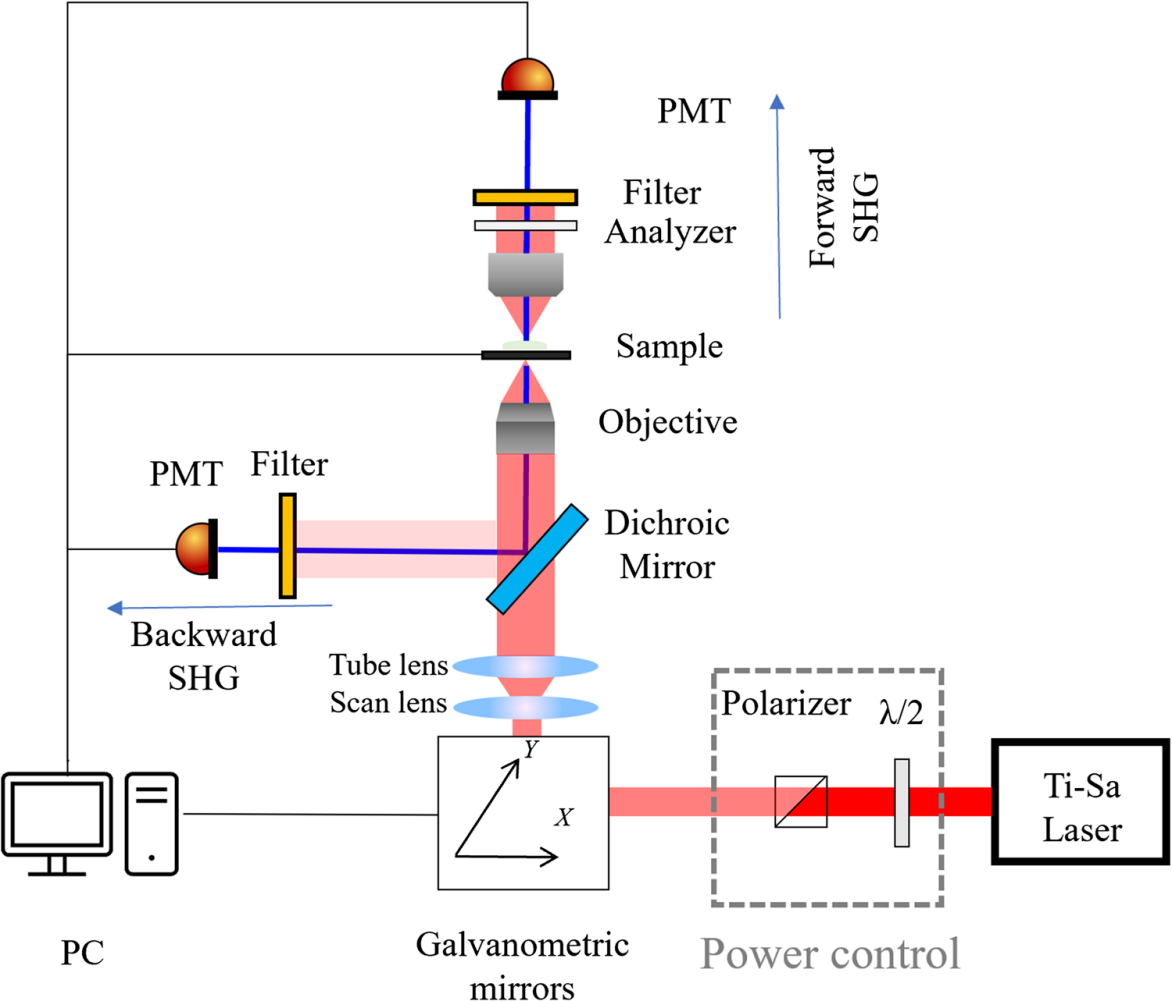
Typical SHG microscopy setup, with source, power control unit, scanning system, objective lens, and detectors. Detectors are connected to a PC that controls the microscope and synchronizes laser scanning with signal acquisition using a detector which is typically a photo multiplier tube (PMT)

In the context of microscopy, 2PEF and SHG present many technical similarities, which allows to combine them easily and efficiently in the same instrument. A typical implementation of a modern SHG microscope, obtained after many experimental setup iterations, is depicted in Fig. 1.

**Fig. 2:**
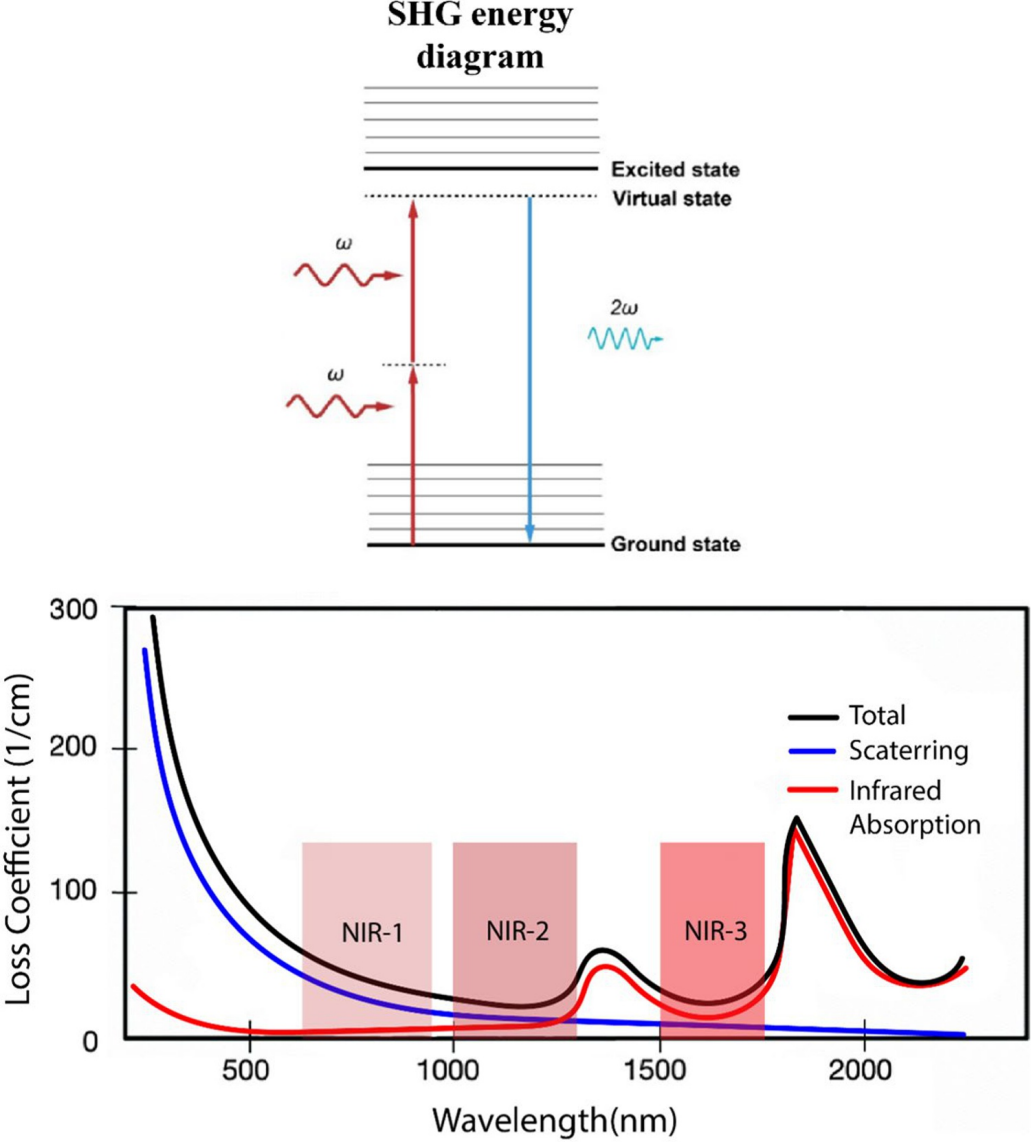
Top: energy level diagram of SHG. Two incident photons interact with the molecules (harmonophores) through virtual states, leading to the generation of a photon at 2*ɷ*, exactly twice the input frequency (*ɷ*). SHG is a parametric process, and no energy transfer occurs. Reproduced under CC BY 4.0 from Borile et al. (2021). Bottom: Absorption spectrum of the human skin, indicating 3 possible transparency windows. Adapted with permission from Hemmer et al. (2013)

Since the obtained imaging depth depends on the excitation wavelength (Helmchen and Denk 2005; Hoover and Squier 2013), the employed laser is traditionally in the NIR-I region (700–1000 nm) (Campagnola 2011) to minimize absorption from biomaterials (water, hemoglobin) (Hemmer et al. 2016). It is worth noting that other optical “windows” matching this criterion are available, as indicated in Fig. 2. Using longer wavelengths, e.g., NIR-2 (1000–1300 nm), allows to limit scattering and hence to increase the penetration depth in the tissues (Helmchen and Denk 2005; Hoover and Squier 2013), however at the expense of a reduced spatial resolution. Despite the higher penetration depth provided by longer wavelengths, it has been shown that, at least for imaging collagenous tissues, longer wavelengths result in lower SHG signal as the hyperpolarizability tensor decreases (Hall et al. 2014). Therefore, shorter wavelengths should still be favored for performance. Besides that, the use of long wavelength lasers (1230 nm), such as Cr:forsterite lasers, provides the opportunity to simultaneously perform SHG and third harmonic generation (THG) microscopy in the visible range, avoiding the UV absorption of biological samples (Chu et al. 2001).

To favor the efficient generation of the non-linear optical signal, the typical pulse duration is about 100 fs at a repetition rate of a few tens of MHz (Hoover and Squier 2013). High numerical aperture (NA > 1) objectives are used to tightly focus the light on the sample and spatially concentrate laser pulse energy (Campagnola and Loew 2003). For thin samples, where the light can be detected in the forward direction (see Fig. 1), a high numerical aperture condenser is added to efficiently collect the light (Cox 2011). Both modalities (2PEF and SHG) present a quadratic dependence of the generated signal to the input laser intensity (Millard et al. 2003), leading to an intrinsic three-dimensional spatial resolution due to the signal generation being confined in the focal volume (Chen et al. 2012).

Despite these similarities, SHG and 2PEF techniques are based on fundamentally different processes. In SHG, the frequency conversion is achieved through virtual states without a net transfer of energy to the system (Fig. 2). This contrasts with 2PEF which involves population transfer from the electronic ground state to excited electronic states. These different origins lead to radically different, and often complementary properties that explain the rising popularity of SHG microscopy.

#### SHG microscopy of endogenous proteins

Second-order non-linear processes, such as SHG, can be efficiently described through an anharmonic oscillator model in which a non-linear restoring force is generated by the molecular potential. At the molecular level, SHG originates from the hyperpolarizability of peptide bonds in collagen and tubulin, usually considered as single SHG emitters (Gusachenko et al. 2012). Indeed, an electric field oscillating at a high frequency and reaching an harmonophore will repeatedly pull the electrons back and forth, leading to the induction of a molecular dipole (Mohler et al. 2003; Chen et al. 2012; Boyd 2019):

1 $${\varvec{p}}={{\varvec{p}}}^{\left(0\right)}+ \alpha {\varvec{E}}+\beta \mathbf{E}\mathbf{E}+\gamma \mathbf{E}\mathbf{E}\mathbf{E}+$$

where *α* is the polarizability of electrons of the peptide bond, ***E*** the incident electric field and *β* and *γ* the hyperpolarizabilities of the first and second order, respectively. The first term ***p***^**(0)**^ is the permanent dipole of the molecule. The second term corresponds to the linear response, the third one defines second-order non-linear interactions, such as sum and difference frequency generation (Boyd 2019), and the fourth term describes third-order non-linear effects (e.g., two-photon absorption (Chen et al. 2012), third harmonic generation (Squier et al. 1998), Kerr effect (Stolen and Ashkin 1973), self-phase modulation (Shimizu 1967), cross-phase modulation (Islam et al. 1987), and stimulated Raman scattering (Houle et al. 2017)).

As a degenerate case of sum-frequency generation, SHG arises from the third term in Eq. 1. Molecules capable of emitting SHG are characterized by a high hyperpolarizability *β*, which strongly depends on their symmetry. Indeed, in the case of a molecule having a center of symmetry, elements contributing to the molecule’s hyperpolarizability cancel each other, preventing SHG formation. More generally, the generation of even harmonics is only possible in non-centrosymmetric materials.

**Fig. 3:**
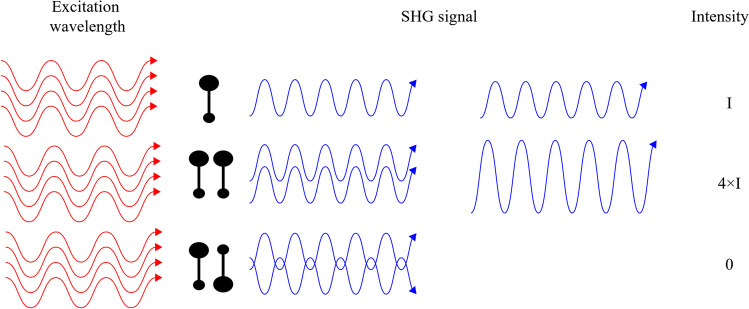
Comparison of the SHG signal from a single dipole (upper row) to the SHG from two parallel (central row) and anti-parallel dipoles (bottom row). Adapted from Bancelin (2014)

The coherent nature of SHG implies that the signal results from interferences of individual contributions of harmonophores. Figure 3 illustrates the case with simple dipoles, separated by a distance negligible with respect to the wavelength of the incoming light wave. When the electric fields emitted by the two dipole moments are in phase and thus constructively interfere, the resulting SHG is coherently added (central row). In contrast, there is destructive interference when the dipole moments have opposite directions and the SHG signal vanishes (Boyd 2019) (bottom row).

At macroscopic scale, SHG is described by the non-linear susceptibility ${\chi }^{\left(2\right)}$, which results from the coherent summation of the individual hyperpolarizabilities of all harmonophores within a volume. The relation between the molecular and macro-molecular non-linear response is given by (Campagnola and Loew 2003; Mohler et al. 2003):

2 $${\chi }^{\left(2\right)}={N}_{S}<\beta >$$

where ${N}_{S}$ is the density of molecule $S$ and < *β* > is the orientational average of the first hyperpolarizability (Boyd 2019). For SHG to occur, at this scale, the medium should exhibit a ${\chi }^{\left(2\right)}\ne 0$ (Campagnola and Loew 2003; Chen et al. 2012), which only happens for non-centrosymmetric macro-molecular organization.

Consequently, to perform SHG microscopy in biological samples, the tissue must present a non-centrosymmetric structure both at the molecular scale ($\beta \ne 0$) and at the macro-molecular level ($<\beta >\ne 0$) as well as a high density of harmonophores. Interestingly, this constraining origin of the signal can be exploited as a contrast enhancing mechanism, since it makes the occurrence of the SHG signal highly specific to only a few biological entities, with collagen as a prime example. SHG can thus act as a unique probe of the multiscale distribution of molecules within the sample.

#### Properties of the SHG signal

Before discussing the properties of the SHG signal, one should have a closer look at the hyperpolarizability and second-order non-linear susceptibility.

In the general case *β*, and therefore ${\chi }^{\left(2\right)}$, are third order tensors with 27 components (${\chi }_{\mathrm{ijk}}^{(2)})$. However, depending on the symmetry of the molecules, the number of non-zero and independent components can be reduced. In this review, we will assume that the Kleinman symmetry condition holds true (Boyd 2019), which requires that the excitation and emission wavelength must be far from resonance, which is the case in most biological samples (e.g., collagen) (Golaraei 2018). Under this assumption, the last two indices of ${\chi }_{\mathrm{ijk}}^{(2)}$ can be freely permuted. Thus, we can regroup the two last indices (jk*)* into a single index *l* and introduce the new tensor:

3 $${d}_{il}=\left[\begin{array}{cccccc}{d}_{11}& {d}_{12}& {d}_{13}& {d}_{14}& {d}_{15}& {d}_{16}\\ {d}_{21}& {d}_{22}& {d}_{23}& {d}_{24}& {d}_{25}& {d}_{26}\\ {d}_{31}& {d}_{32}& {d}_{33}& {d}_{34}& {d}_{35}& {d}_{36}\end{array}\right]=\frac{1}{2}{\chi }_{\mathrm{ijk}}^{(2)}$$

Note that with the Kleinman symmetry and the permutation, not all 18 components in the matrix are independent (${d}_{12}={d}_{26}$ and ${d}_{14}={d}_{25}$). Considering only the second-order effect in Eq. 1, the dipole momentum induced by the incident laser is given by the following:

4 $$\left[\begin{array}{c}{p}_{x}^{(2)}(2\omega )\\ {p}_{y}^{(2)}(2\omega )\\ {p}_{z}^{(2)}(2\omega )\end{array}\right]\propto \left[\begin{array}{cccccc}{d}_{11}& {d}_{12}& {d}_{13}& {d}_{14}& {d}_{15}& {d}_{16}\\ {d}_{21}& {d}_{22}& {d}_{23}& {d}_{24}& {d}_{14}& {d}_{12}\\ {d}_{31}& {d}_{32}& {d}_{33}& {d}_{34}& {d}_{35}& {d}_{36}\end{array}\right]\left[\begin{array}{c}{E}_{x}^{2}(2\omega )\\ {E}_{y}^{2}(2\omega )\\ {E}_{z}^{2}(2\omega )\\ {2E}_{y}(\omega ){E}_{z}(\omega )\\ {2E}_{x}(\omega ){E}_{z}(\omega )\\ {2E}_{x}(\omega ){E}_{y}(\omega )\end{array}\right]$$

Equation 4 shows that the polarization of the input laser beam is of utmost importance since it is directly related to the tensor elements (Chu et al. 2004; Pavone and Campagnola 2013) and therefore largely determines the formation of SHG signal.

In the following case, we will use collagen as an example, but this can also be extended to other materials by considering their specific symmetry. A collagen fibril presents a cylindrical symmetry. We will make two assumptions: first that the Kleinman symmetry is applicable (Golaraei 2018) and secondly that the chiral components of the tensor can be neglected since we do not take the out-of-focus orientation into account (Golaraei 2018). In this condition, the non-linear susceptibility tensor has only two independent components which are ${\chi }_{\mathrm{xxx}}$ and ${\chi }_{\mathrm{xyy}}$, where *x* is the fibrillar axis. Thus, considering that the input laser is linearly polarized and propagates along the *z*-axis, the SHG intensity in every pixel of an image is as follows:

5 $${I}_{\mathrm{SHG}}\left(\theta ,\mu \right)=A+B\mathrm{cos}\left(2\mu -2\theta \right)+C\mathrm{cos}(4\mu -4\theta )$$

where $\mu$ is the polarization angle with respect to the *x*-axis, $\theta$ the azimuthal angle of the fibril (see schematic in Fig. 8) with the *x*-axis, and A, B, C are coefficients depending on the harmonophore concentration and arrangement (Odin et al. 2008). Therefore, varying the incident polarization strongly affects the SHG intensity. This, in turn enables to probe macro-molecular organization of harmonophores within the focal volume (James and Campagnola 2021). Alternatively, the use of circularly polarized excitation light ensures that all molecules respond similarly, regardless of their in-plane orientation (Chen et al. 2012).

Since SHG is a coherent process, the phase plays a key role in the signal formation, from the molecular to the macro-molecular scale. This can be clearly highlighted considering the case of SHG from bulk media. A complete description of the formalism in this case can be found in (Boyd 2019). In brief, considering an incident laser beam with fixed polarization and propagation direction, and assuming the slowly varying envelop approximation, the SHG intensity can be expressed as follows:

6 $${I}_{\mathrm{SHG}}\propto {\left|\uppsi \right|}^{4}{L}^{2}{\mathrm{sinc}}^{2}\left(\frac{\Delta kL}{2}\right)={{I}_{\mathrm{in}}}^{2}{L}^{2}{\mathrm{sinc}}^{2}(\frac{L}{{L}_{c}})$$

where $\psi$ is the complex amplitude of the incident beam, *I*_in_ is the intensity of the incident laser beam, *L* is the length over which SHG occurs in the medium, $\Delta k=2{k}_{\omega }-{k}_{2\omega }$ is the phase mismatch between the excitation and the emitted light (expressed as the difference of wave-vectors) and ${L}_{c}=2/\Delta k$ is the coherence length. Consequently, when the phase-matching condition $\Delta k=0$ is fulfilled, the SHG intensity directly scales with the square of the input laser intensity and with the square of *L*. However, if $\Delta k\ne 0,$ the SHG intensity reaches a maximum value after an interaction length of ${\pi L}_{c}/2$. In that case, if the interaction length *L* is any longer in the material, the SHG intensity oscillates between zero and the maximum value over a spatial period of ${2\pi L}_{c}$.

In biological samples, the phase-matching condition is rarely fulfilled, leading to a directionality of the SHG signal. However, $\Delta kL$ is nearly equal to zero for the forward direction since the length of interaction is small compared to ${L}_{c}$ (few microns), due to the tight focusing. In backward direction, this is not the case since $\Delta k$ is much larger and the coherence length is much shorter (a few tens of nanometer). This explains why “pure” backward SHG is always very weak. This effect will be further discussed in “Advanced SHG microscopy.”

#### SHG microscopy in biological samples

One of the most ubiquitous proteins in body tissue that can be imaged using SHG microscopy is collagen, a family of proteins found in most connective tissues. At the molecular scale, collagen consists of three *α*-chains, called tropocollagen, which are hydrogen bonded to each other (Chen et al. 2012). In some collagen types (mostly I and II) these triple helices spontaneously self-assemble into highly organized collagen fibrils (Cox 2011) leading to very strong SHG signals (Campagnola et al. 2002). In contrast, non-fibrillar collagen (e.g., type IV), which forms sheets in basal laminae (Cox 2011), cannot be visualized with SHG microscopy (Cox et al. 2003).

**Fig. 4:**
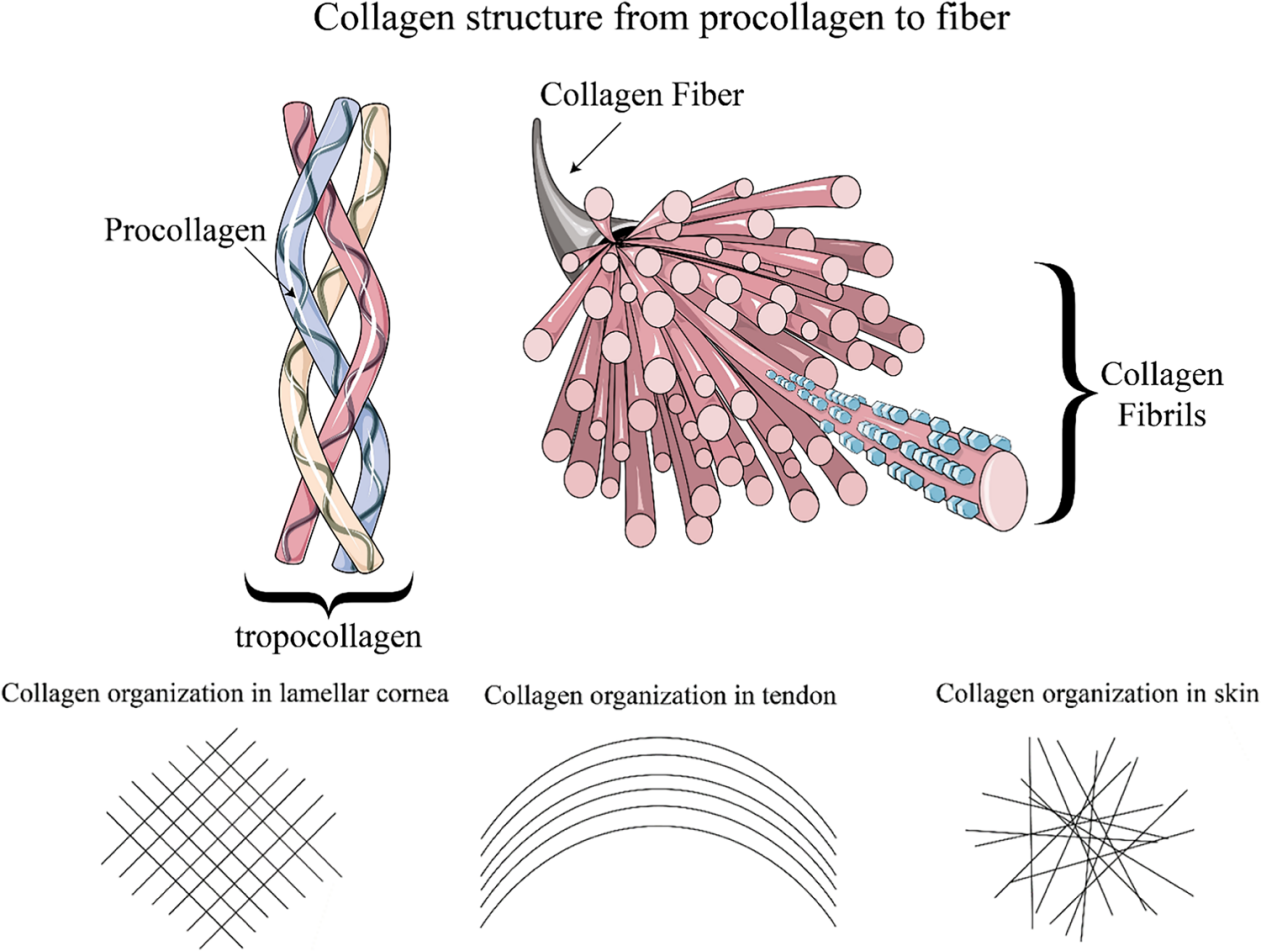
Top: Hierarchical structure of collagen. modified under CC BY-SA 3.0 from Laboratoires Servier (2019). Bottom: Collagen organization in different biological entities (Morishige et al. 2011; Rivard et al. 2014; Mostaço-Guidolin et al. 2017)

The first demonstration of SHG microscopy in biological tissue has been performed using rat-tail tendons by Freund and co-workers (Freund and Deutsch 1986). In this tissue, collagen type I forms a highly organized multiscale structure as depicted in Fig. 4. SHG microscopy has been used to image achilles tendon and fascia (Légaré et al. 2007). It has also found application to monitor the healing process of tendons (Hase et al. 2016).

**Fig. 5:**
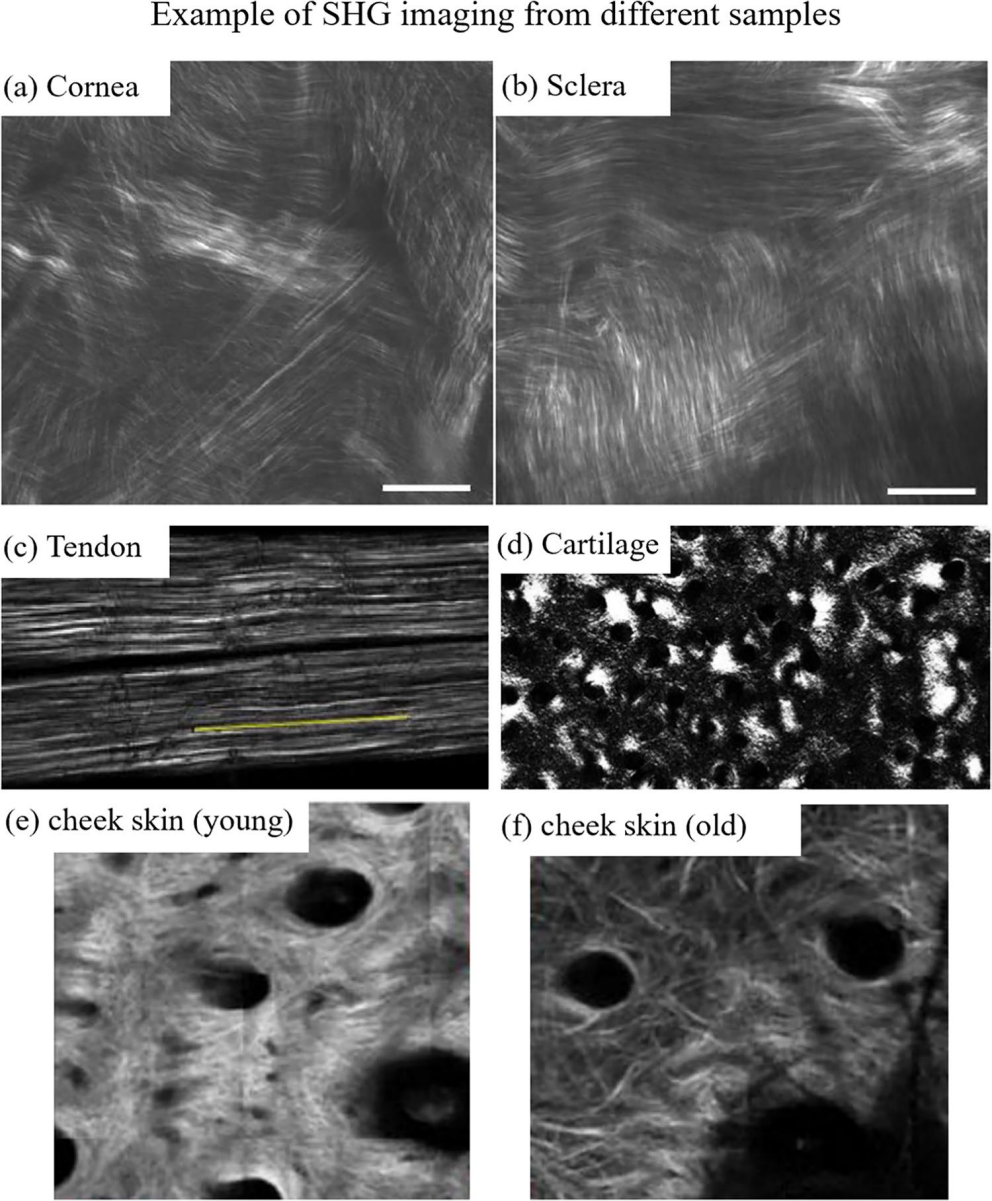
Examples of SHG images for various biological samples. SHG images from (**a**) cornea and (**b**) sclera, the scale bar is 20 μm. Extracted from Han et al. (2005). (**c**) Tendon (500 × 150 µm), extracted with permission from Rivard et al. (2014). (**d**) Cartilage (350 × 200 µm), extracted with permission from Couture et al. (2015). SHG image of skin in young (**e**) vs. old age (**f**) (1.6 × 1.6 mm), extracted with permission from Ogura et al. (2019)

The eye is one prominent example containing—mostly—collagen type I in two different components: the cornea and the sclera (Bueno et al. 2016), which hence can be visualized using SHG microscopy. An example is shown in Fig. 5(a) and (b). Within the cornea, the collagen is arranged in a lamellar configuration contributing to corneal transparency (Bueno et al. 2016) while in the sclera, collagen fibrils are randomly packed and highly scattering (Teng et al. 2006). Tendon and cartilage are two other tissues that have been well-studied using SHG microscopy with examples shown in Fig. 5(c) and (d) (Rivard et al. 2014; Couture et al. 2015). The skin is another biological component that has been imaged by SHG microscopy. As an example, a recent study by Ogura et al. compared skin samples from humans in their young, middle, and old age, reporting that the concentration of thick collagen declines with age (Ogura et al. 2019) (see Fig. 5(e) and (f)).

In addition, SHG microscopy has been proven useful to image cartilage and bones (Yeh et al. 2005; Houle et al. 2015), which are composed of collagen type II. This opened avenues to investigate damages of the extracellular matrix that can result in loss of structure integrity, which leads to various pathologies such as osteoarthritis. Many pathologies such as cancer can be investigated and characterized using these techniques but are beyond the scope of this review. Extensive details on these applications can be found in the literature (Stoller et al. 2003; Chen et al. 2012; Kwan 2013; Preston et al. 2022).

Beyond collagen, other biological structures have been investigated by means of SHG microscopy. Myosin is a motor protein involved in a wide variety of functionalities, such as muscle contraction, or cellular movements that are largely influenced by the interaction between actin and myosin (Lodish et al. 2000). Therefore, the visualization of the myosin structure is bound to increase our understanding of fundamental mechano-cellular mechanisms. Mohler et al. first observed a strong SHG signal in mouse muscle and then confirmed in *C. elegans* that the signal arises from the heavy-chain B of myosin (Mohler et al. 2003). Studies combining SHG microscopy and 2PEF revealed enlarged lysosomes in Pompe disease and provided advanced characterization of the morphology of cardiomyocytes (Ralston et al. 2008; Wallace et al. 2008). A combination of SHG and coherent anti-Stokes Raman scattering has also been used to study muscle structure (Pfeffer et al. 2011). More recently, wide-field SHG was applied for imaging muscle contractions, which will be briefly discussed in “Advanced SHG microscopy” (Zhao et al. 2019)."

Microtubules (MTs) are another key element that can be imaged using SHG microscopy (Kwan 2013), allowing fascinating studies in neurosciences and developmental biology. “SHG and enhanced SHG in neurons” is specifically dedicated to present the recent advances of SHG microscopy for MTs studies.

**Fig. 6:**
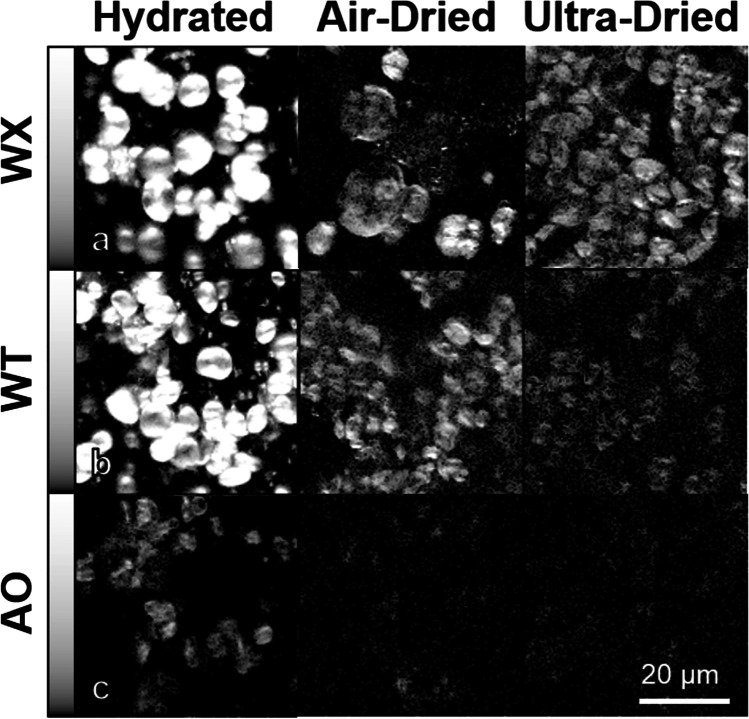
SHG imaging of three types of barley starch granules in different hydration states. **a** WX (waxy barley with only amylopectin) exhibits a very high SHG intensity even in ultra-dry conditions. **b** WT (wild-type barley with ~ 30% amylose content) SHG signal is dimmer than in panel **a** but still detectable. **c** AO (amylose only barley) has the lowest SHG signal intensity among the three, which is barely detectable in ultra-dried condition. Extracted from Cisek et al. (2015)

Finally, beyond the study of body tissues, another application of SHG microscopy is for imaging polysaccharide chains in plants and notably in starch. Starch plays an important role in energy storage for plants and represents a major source of food for humans. In 2005, Cox et al. reported on SHG signal from cellulose and starch, which can be explained by their highly crystalline structure (Cox et al. 2005). However, while the starch SHG signal can easily be detected at low input powers, acceptable for biological tissue imaging, cellulose was found to be a weak SHG emitter. In the same study, the authors suggested that the origin of the SHG signal in starch granules is from two polysaccharides, namely amylopectin and amylose. By performing SHG and polarization-resolved SHG microscopy on starch from rice and rice flour, Zhuo et al. demonstrated that the SHG emitter in starch were only amylopectin and not amylose (Zhuo et al. 2010). Building upon this study, Cisek et al. examined barley and found that wild-type amylopectin crystals generate higher SHG signal due to their long-range order (Cisek et al. 2015). On the other hand, structures containing amylose have much lower crystalline order leading to much lower SHG emission (~ 20 times less) (Cisek et al. 2015). Moreover, the hydration state strongly affects the SHG intensity of starch granules (Cisek et al. 2014, 2015). Hydrated granules have a higher SHG intensity (Fig. 6) due to the more ordered crystalline hydroxide and hydrogen bonds forming long-range orders, whereas ultra-dry structures have a more disordered structure (Cisek et al. 2015).

### Advanced SHG microscopy

Beyond the imaging capability, the coherent and tensorial nature of the SHG process enables us to extract additional information about the sample. This section will outline the main approaches that have been developed over the years and applied to various biological investigations.

#### Forward over backward second harmonic generation (F/B-SHG)

Forward over backward (also called “directional”) SHG microscopy is a method that takes full benefit from the directionality of the SHG radiation pattern. For complete description, we suggest ref. Chen et al. (2012).

**Fig. 7:**
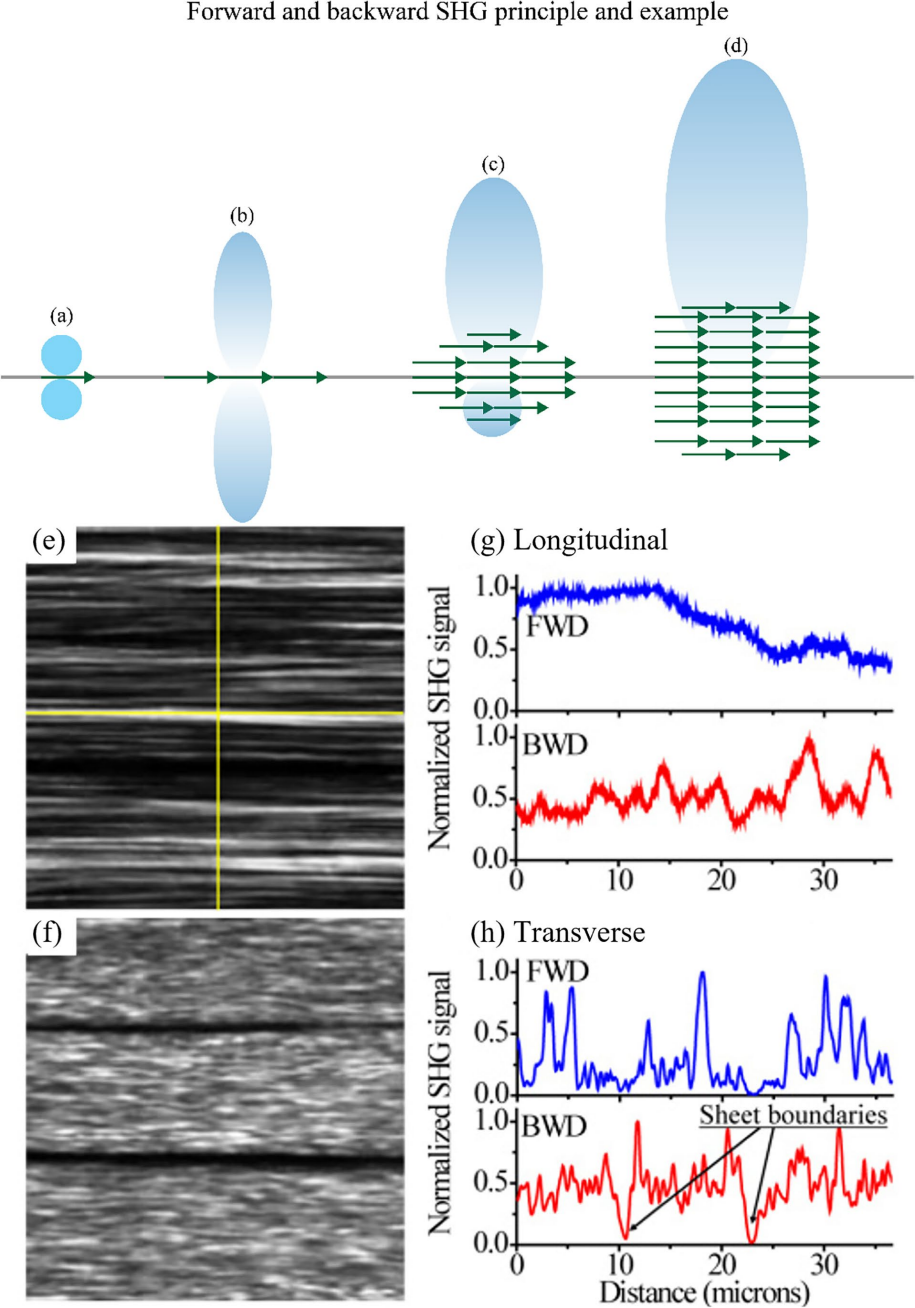
Radiation pattern for different dipole configurations in the focal volume. (**a**) A single dipole, indicated by the green arrow, creates equal F-SHG and B-SHG (F/B = 1). (**b**) Several dipoles in the same plane also create the same amount of SHG signal in the forward and backward direction (F/B = 1). (**c**) The coherent contribution of many induced dipoles packed in the optical direction will generate a strong forward SHG signal and a weak backward SHG signal (F/B > 1). (**d**) For a bulk material, only a strong forward SHG signal is present with a negligible amount of backward SHG. Adapted under CC BY-SA 4.0 from BP-Aegirsson. Forward (**e**) and backward (**f**) SHG images of fascia. Panels (**g**) and (**h**) respectively represent longitudinal and transverse intensity profiles (with respect to the fibrillar axis (horizontal axis)), as depicted by the yellow crosshair in (**e**), taken in forward (blue) and backward (red) direction. In the backward direction, the sheet boundaries are easier to spot than in the forward SHG image. Extracted from Rivard et al. (2010)

Because it is a coherent process, SHG conserves the spatial coherence of the excitation. The harmonic photons are scattered over an angle smaller than the Gaussian beam angle of the excitation. As previously mentioned, (see “Properties of the SHG signal”), perfect phase-matching is never met in SHG microscopy. The coherence length for forward SHG (F-SHG) is a few microns in most materials, which is enough for a consistent phase-matching within a focal volume (not accounting for Gouy phase shift effects though). In contrast, the coherence length for backward SHG (B-SHG) is only a few tens of nanometers in most materials, which means that the B-SHG signal is always poorly phase matched. In practice, the B-SHG signal is always smaller than the F-SHG one, reaching equality only when one dipole or an extremely thin structure is excited along the propagation direction (Fig. 7(a–b)). The F-SHG contribution becomes much larger when many dipoles are stacked along the focal volume (Fig. 7 (c–d)), which is usually the case in biological samples. Importantly, since the B-SHG signal is usually weak, it should not be confounded with backscattered F-SHG signal. Indeed, since most biological samples are highly scattering, a significant part of the F-SHG gets scattered or reflected towards the backward direction after its generation (see Fig. 7) (Légaré et al. 2007).

Figure 7 shows an example of F/B-SHG images. The F-SHG signal along the fibrils direction (longitudinal) remains exceptionally smooth (Fig. 7 (e)), revealing that fibril bundles form domains of constant ${\chi }^{\left(2\right)}$ that can lead to a better fulfillment of the phase-matching condition. On the contrary, in the direction perpendicular to the fibrils (transverse), multiple/different ${\chi }^{\left(2\right)}$ domains boundaries are encountered, leading to rapid changes in phase-matching and high modulation of the F-SHG signal (Fig. 7(f) and (h)). This is in agreement with the conclusion of Freund and Deutsch (Freund and Deutsch 1986) as well as with the measurements of Parry and Craig using electron microscopy (Parry and Craig 1977). It is important to highlight that the dark lines in the collagen sheets in the forward image (e) are not due to the lack of collagen fibrils, but due to long ${\chi }^{\left(2\right)}$ domains whose macro-molecular structure results in poor phase-matching, leading to low signal along the full length of the domain. In contrast, since the coherence length in B-SHG is much shorter, the arrangement of the domains has almost no impact on the amount of signal generated. Therefore, the backward image is mostly uniform along the whole tissue.

Effectively, due to the different coherence lengths for F- and B-SHG, the F-SHG signal images display ordered structures whose size are on the order of *λ*_SHG_ (SHG wavelength), while smaller or more random structures are better revealed in B-SHG, both directions providing complementary images (Chen et al. 2012). In the case of collagen, the F/B ratio increases either with the fibrils’ diameter or when fibrils of the same polarity are bundled (Brown et al. 2013; Rivard 2016). Since this ratio is usually averaged over the whole field-of-view, it quantifies the average size and global arrangement of the collagen bundles in the sample (Chen et al. 2012).

#### Polarization-resolved second harmonic generation (P-SHG)

**Fig. 8:**
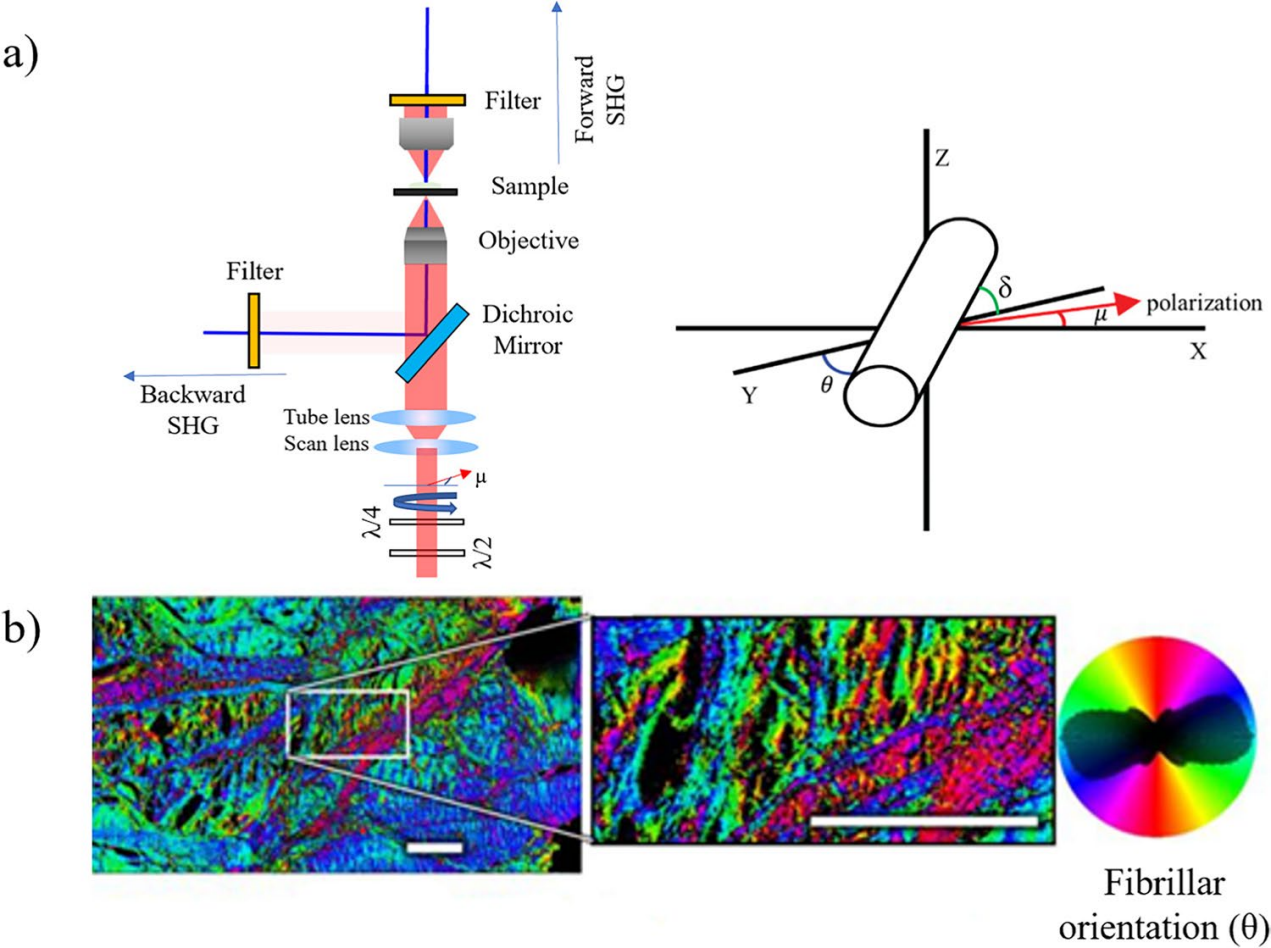
**a**) Schematic of a typical P-SHG microscope with sample in focus. A half-wave plate (*λ*/2) and a quarter-wave plate (*λ*/4) are used to control the pump polarization state. Adapted from Teulon et al. (2015). **b**) Collagen fibril orientation in adult horse specimens measured by P-SHG. In this study, the maturation of meniscal collagen was studied in young and adult horses using P-SHG. Extracted under CC BY 4.0 from Pinsard et al. (2019b)

P-SHG couples the benefit of SHG microscopy (high specificity and contrast) and polarimetry (sensitivity to molecular alignment). Usually applied to collagen, it can reveal more accurately the complex hierarchical structures of fibrils in the image plane. One of its first demonstrations has been realized on rat-tail tendon fascia by Stoller and co-workers in 2002 (Stoller et al. 2002a). Acquiring different linear polarization scans in the axial and transverse plane, they identified that the SHG signal was highly affected by the polarization of the input laser light source, allowing the determination of the orientation of collagen fibrils. Figure 8 provides an example of a P-SHG system with an application example from an adult horse meniscal collagen.

To characterize the collagen fibrils’ orientation, various parameters can be measured such as the average in-plane azimuthal angle *θ*, in every pixel, and the anisotropy parameter *ρ* as indicated in the upper right corner of Fig. 8 (Pinsard et al. 2019b):

7 $$\rho = {\sqrt{\frac{{I}_{\parallel }}{{I}_{\perp }}}=\rho }_{0}{\mathrm{cos}}^{2}\delta +3{\mathrm{sin}}^{2}\delta$$

where ${I}_{\parallel }$ (resp. ${I}_{\perp }$) is the SHG intensity when the incident polarization is parallel (resp. orthogonal) to the fibril, *δ* is the out-of-plane tilt angle of the fibril and ${\rho }_{0}=\rho \left(\delta =0\right)={d}_{33}^{\left(2\right)}/{d}_{31}^{\left(2\right)}$ is the anisotropy parameter for no tilt (i.e., $\delta =0$) (Psilodimitrakopoulos et al. 2009).

Alternatively, for no out-of-plane tilt, the measure of the “anisotropy parameter” *r* (Campagnola and Loew 2003) can be used:

8 $$r=\frac{{I}_{\parallel }-{I}_{\perp }}{{I}_{\parallel }+{2I}_{\perp }}$$

Here *r* = 0 corresponds to an isotropic orientation and *r* = 1 to the fully aligned case. In practice, $r$ ~ 0.7 in highly aligned collagen tissue such as tendon (Campagnola and Loew 2003). Other parameters such as the entropy of orientation (Ducourthial et al. 2019) or the orientation index (O.I.) (Hristu et al. 2017) can also be extracted from P-SHG and some studies also reported the variance of the contrast-per-pixel as meaningful metrics for P-SHG (Stanciu et al. 2017).

One efficient approach to extract information from P-SHG is based on Fourier transform analysis. In that case, only the input polarization is rotated using half- and quarter-wave plates (Latour et al. 2012) (Fig. 8). Afterwards, the relevant information can be retrieved from the P-SHG images using an analysis based on the Fourier transform of the measured intensity with respect to the input polarization angle. This method is applicable to B-SHG and backscattered F-SHG signal, making it particularly well suited for thick in vivo samples (Latour et al. 2012).

A more advanced modality, called PIPO (polarization in–polarization out) (Tuer et al. 2012), introduces an additional rotating analyzer in the detection path, in order to extract the asymmetry of fibrils distribution $\varsigma$, in complement to the anisotropy *ρ*:

9 $$\varsigma =\frac{<\mathrm{sin\;}\delta >}{<\mathrm{cos}\;\delta >}$$

where < … > is the weighted average.

In the past, P-SHG emerged as a powerful tool for biomedical applications, especially to probe protein structure. Previously, cryo-EM (Binshtein and Ohi 2015) and X-ray crystallography (Shi 2014) were the tools of choice for this study but both methods require complex and intensive sample preparation, preventing their use on live dynamic samples let alone on living animals (Kaneshiro et al. 2019). Alternatively, to investigate structural dynamics of proteins, other methods have been used, such as nuclear magnetic resonance and Forster resonance energy transfer (FRET), which are more readily available but have lower spatial resolution and low sensitivity (Kaneshiro et al. 2019). In contrast, P-SHG can be applied in pristine samples and does not rely on complex and expensive devices for analysis since it only requires adding a few optical components to a regular SHG microscope (Kaneshiro et al. 2019). Recently, P-SHG has been used to study collagen alteration in aging (Miler et al. 2021), keratoconic cornea (Raoux et al. 2021), and collagen structure alteration in lung cancer (Golaraei et al. 2020). Note that high precision control of the polarization can be achieved using electro-optical modulators (Stoller et al. 2002b).

#### Circular dichroism second harmonic generation (CD-SHG)

**Fig. 9:**
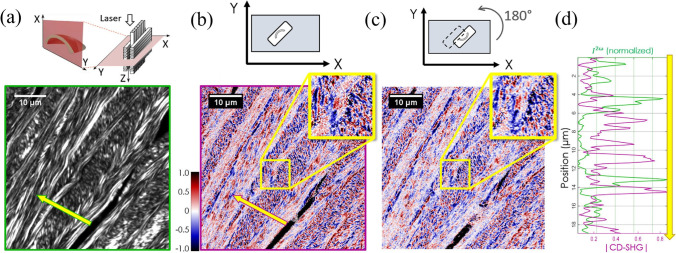
Example of CD-SHG applied in the transverse imaging of a human cornea. (**a**) Schematics and SHG intensity image of the cornea. Panels (**b**) and (**c**) show the CD-SHG imaging of the same region of the sample in two different configurations. As it is evident, the CD-SHG sign in both configurations is the same for almost 80% of the imaged pixels. Lastly, in panel (**d**), the SHG intensity profile (in green) and the CD-SHG absolute value (in magenta) are plotted along the yellow arrow shown in panels (**a**) and (**b**). Modified from Schmeltz et al. (2020)

Beyond P-SHG, the use of laser light with left- and right-handed circular polarization (LCP and RCP respectively) allows to extract the so-called circular dichroism SHG (Verbiest et al. 1999; Tuer et al. 2012):

10 $${I}_{\mathrm{CD}-\mathrm{SHG}}=\frac{{I}_{\left(2\omega \right)\mathrm{LCP}}-{I}_{\left(2\omega \right)\mathrm{RCP}}}{\left({I}_{\left(2\omega \right)\mathrm{LCP}}+{I}_{\left(2\omega \right)\mathrm{RCP}}\right)/2}$$

where *I*_CD-SHG_ is obtained from subtracting two SHG images acquired with LCP and RCP, respectively. Just like circular dichroism detected in linear microscopy, CD-SHG requires an optical activity to be non-zero (which is concomitant to a chiral symmetry). Yet, non-linear CD does not mandatorily originate from the interaction between electric and magnetic dipole moments (as for linear CD) but can result from electric dipoles alone (Schmeltz et al. 2019). A recent study demonstrates the use of CD-SHG to investigate and characterize 3D collagen distribution. Indeed, the absolute *I*_CD-SHG_ enables to determine whether the fibrils are oriented in the imaging plane (small *I*_CD-SHG_ values) or out of it (high *I*_CD-SHG_ values) (Pinsard 2020; Schmeltz et al. 2020), and it notably shows great promise in measuring the polarity of out-of-plane collagen fibrils. As an example, Fig. 9 shows CD-SHG and its application in imaging human cornea.

Consequently, CD-SHG shows tremendous potential in pathological tissue diagnosis, for which disordered collagen and 3D remodeling of collagen are critical structures and processes.

#### Stokes vector–based second harmonic generation microscopy

While changing the linear or circular input polarization state and measuring the change in SHG intensity allows to measure linear birefringence and anisotropy of the sample, this does not provide the full polarimetric response of a sample (Mazumder et al. 2012). Indeed, in previously presented methods (“Polarization-resolved second harmonic generation (P-SHG)” and “Circular dichroism second harmonic generation (CD-SHG)”) fully polarized light is used, represented by Jones calculus, and does not consider all states of light, namely incoherence, partially polarized and unpolarized light (Qiu et al. 2012; Mazumder et al. 2012, 2017). Besides the input light state, biological samples are not always well-organized and non-regular arrangements can lead to incomplete polarimetry results. For a complete description of the polarimetric response of the material, Stokes-Mueller matrix formalism is better suited.

The state of polarization of light can be fully characterized through a 4 × 1 Stokes vector $S$:

11 $$S=\left[\begin{array}{c}{S}_{0}\\ {S}_{1}\\ {S}_{2}\\ {S}_{3}\end{array}\right]=\left[\begin{array}{c}{I}_{0}+{I}_{90}\\ {I}_{0}-{I}_{90}\\ {I}_{45}-{I}_{-45}\\ {I}_{R}-{I}_{L}\end{array}\right]$$

where ${I}_{0}$ is the intensity at ${0}^{^\circ }$, ${I}_{90}$ is the intensity at $9{0}^{^\circ }$, ${I}_{\pm 45}$ is the intensity at ${\pm 45}^{^\circ }$ and ${I}_{R}$ and ${I}_{L}$ represent the intensity at right and left polarization states. All the elements of the matrix are between − 1 and + 1, as they are normalized to the value of ${S}_{0}$. From this, vector, we can describe important polarimetric parameters such as the degree of polarization ($\mathrm{DOP}$), the degree of linear polarization ($\mathrm{DOLP}$), and the degree of circular polarization ($\mathrm{DOCP}$) (Qiu et al. 2012; Mazumder et al. 2012):

12 $$\mathrm{DOP}=\frac{{\left({S}_{1}^{2}+{S}_{2}^{2}+{S}_{3}^{2}\right)}^\frac{1}{2}}{{S}_{0}}$$

13 $$\mathrm{DOLP}=\frac{{\left({S}_{1}^{2}+{S}_{2}^{2}\right)}^\frac{1}{2}}{{S}_{0}}$$

14 $$\mathrm{DOCP}=\frac{\left|{S}_{3}\right|}{{S}_{0}}$$

**Fig. 10:**
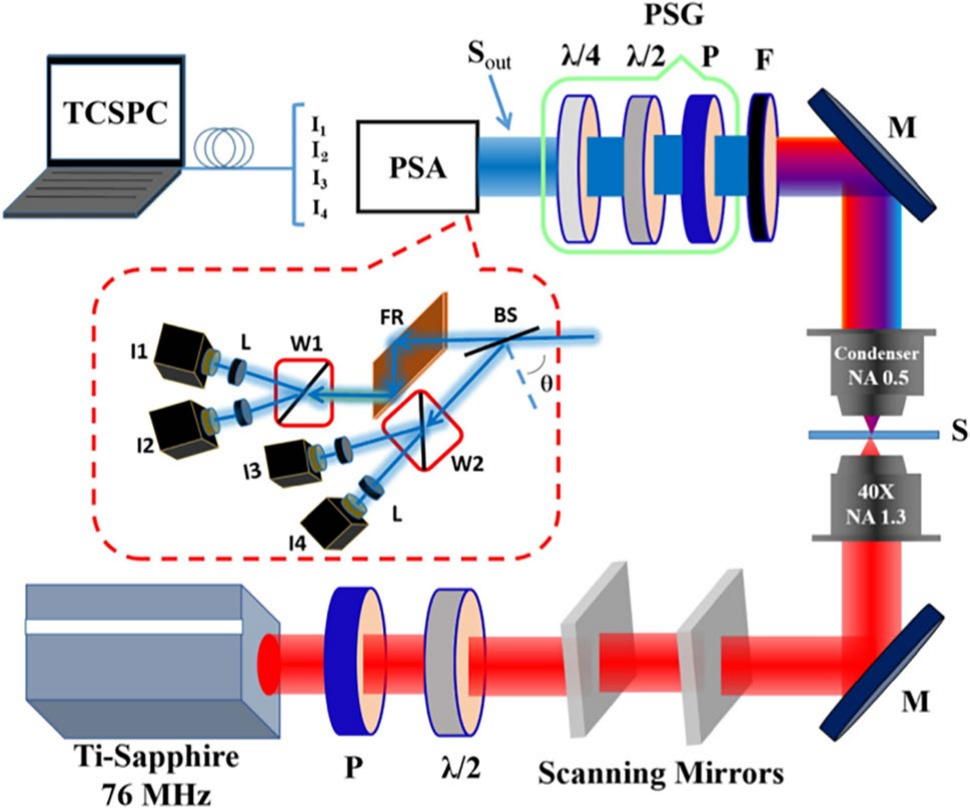
Example of a four-channel-Stokes polarimeter SHG microscopy setup. After the SHG from the sample, the signal passes through a polarization state generator consisting of a polarizer, a half-wave plate, and a quarter-wave plate before passing through a polarization state analyzer comprised of a beam splitter, a Fresnel rhomb, and two Wollaston prisms. It is detected simultaneously by a time-correlated single-photon-counting (TCSPC) system consisting of four detectors. Reproduced under CC BY 4.0 from Mazumder et al. (2012)

**Fig. 11:**
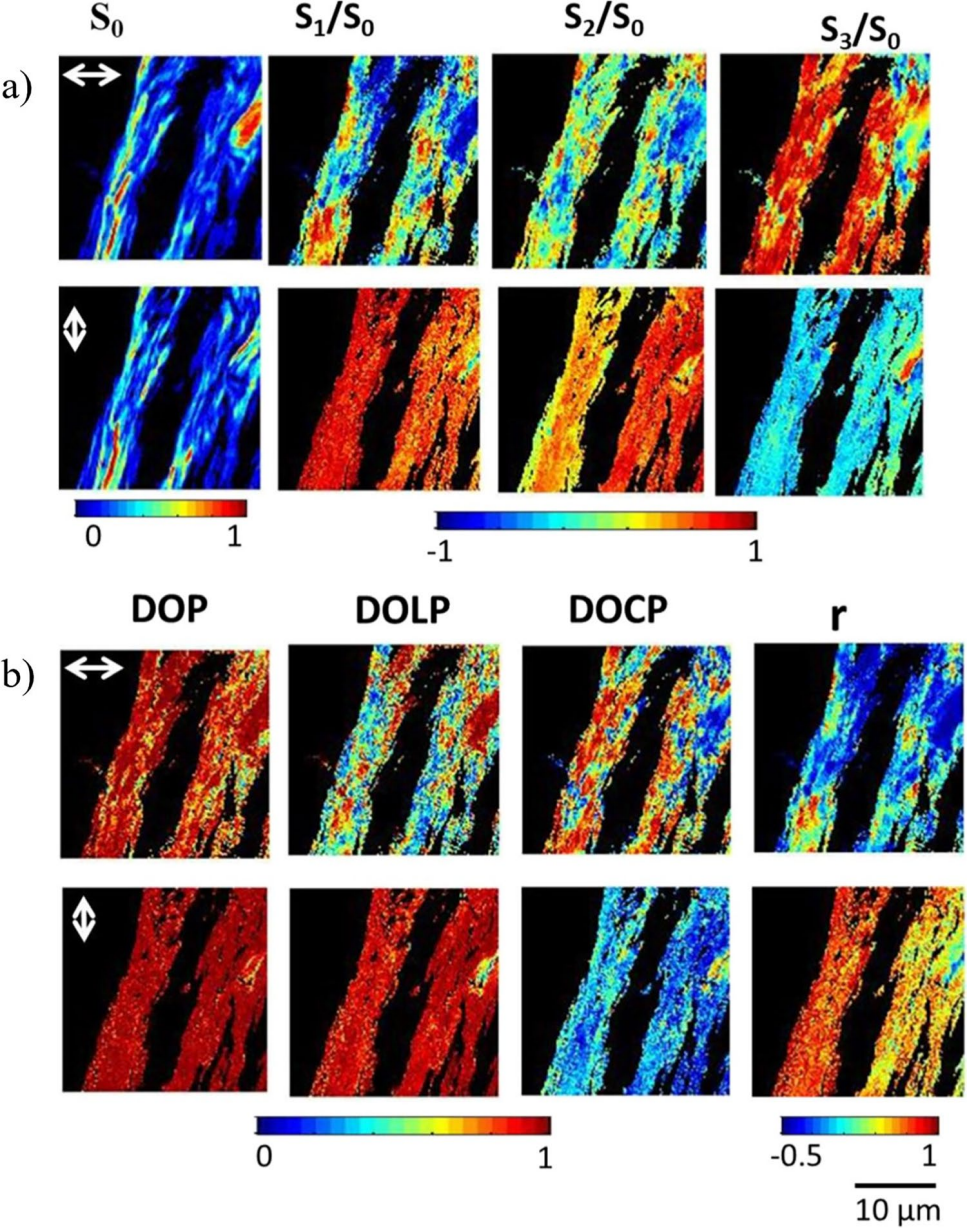
Stokes vector–based SHG microscopy of collagen fibers. Panel **a**) Represents the 2D Stokes vector images of the collagen fibers with vertical and horizontal input polarization. Panel **b**) shows the DOP, DOLP, DOCP, and anisotropy parameter of the collagen fibers. Modified under CC BY 4.0 from Mazumder and Kao (2021)

Stokes vector–based SHG microscopy has been implemented using a four-channel-Stokes polarimeter (Mazumder et al. 2012), as depicted in Fig. 10:

The relation between the output Stokes matrix ${S}_{\mathrm{out}}$ and the four detected intensities is given by the following:

15 $${S}_{\mathrm{out}}={A}_{4\times 4}^{-1}.I={A}_{4\times 4}^{-1}.{\left[{I}_{1},{I}_{2},{I}_{3},{I}_{4}\right]}^{t}$$

where ${A}_{4\times 4}^{-1}$ is the polarimeter instrument matrix and $I$ is composed of the four detected SHG intensities (Mazumder et al. 2012, 2017). This technique has recently been used to characterize collagen fibers in adult mice tails, as shown in Fig. 11:

One of the main drawbacks of this method is its restriction to forward detection configuration, and hence thin samples (Mazumder and Kao 2021). In addition, this method assumes a linear relation between the incoming laser light and the SHG signal and still does not provide a complete polarimetric response of the sample (Kontenis et al. 2016).

A more generalized approach is the double Stokes-Mueller polarimetry method (DSMP). In this method, a complete and model-independent SHG polarimetric response is represented by measuring 36 polarizations at minimum to calculate all observable laboratory frame tensor components (Kontenis et al. 2016). The relationship between the polarization of the output SHG signal and the polarization state of the input laser beam is given by the double Mueller matrix (Kontenis et al. 2016):

16 $${S}_{\mathrm{SHG}}\left(2\omega \right)={M}^{\left(2\right)}{S}_{\mathrm{in}}(\omega )$$

where ${S}_{\mathrm{SHG}}\left(2\omega \right)$ is the 4 × 1 SHG signal Stokes vector at $2\omega$ frequency, ${S}_{\mathrm{in}}(\omega )$ is the 9 × 1 input double Stokes vector describing the state of the two incident photon at $\omega$ frequency and ${M}^{\left(2\right)}$ is the 4 × 9 double Mueller matrix which is dependent on the non-linear susceptibility of the material (Kontenis et al. 2016).

**Fig. 12:**
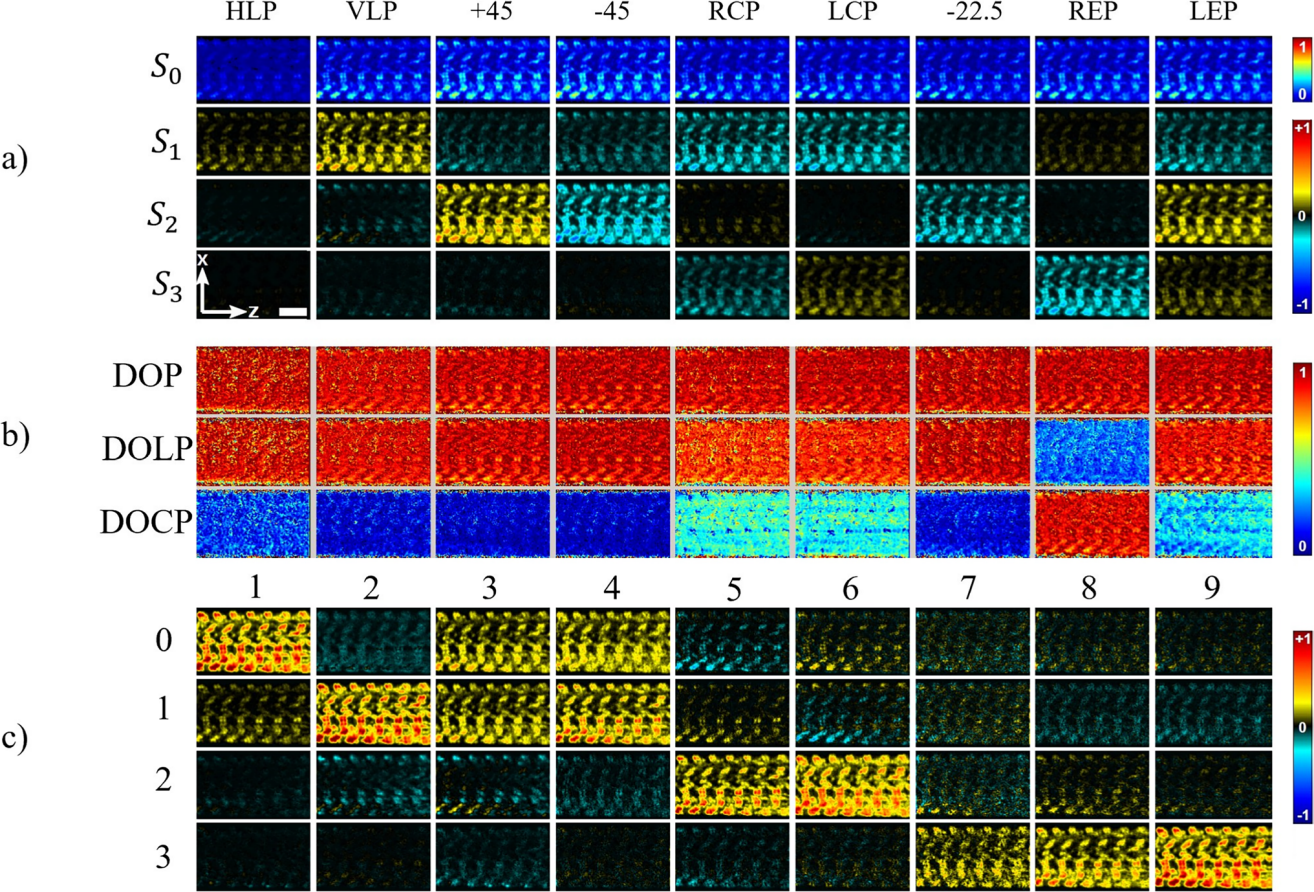
DMSP SHG images of the wall muscle of *Drosophila melanogaster* larva. **a**) Measured Stokes matrix elements. **b**) Maps of DOP, DOLP, and DOCP. **c**) Double Mueller matrix elements normalized to the value obtained for ${\chi }_{\mathrm{ZXX}}^{(2)}$ from imaging. The scale bar is 10 µm. Modified from Kontenis et al. (2016)

A complete characterization requires 9 polarimetric measurements for DSMP: horizontal and vertical linear polarization (HLP and VLP), right-handed and left-handed circular polarization (RCP and LCP), diagonal polarization ($\pm {45}^{^\circ })$, right-handed and left-handed elliptical polarization (REP and LEP) and a linear polarization at $-{22.5}^{^\circ }$. The DOP is then calculated and filtered, for removing the scattering contribution, prior to calculate the double Mueller matrix of the sample. Using the six non-phase matrix elements of the double Mueller matrix, the laboratory frame non-linear susceptibility tensor values can be completely retrieved. In the end, the molecular-frame orientation and non-linear susceptibility tensor ratios can be obtained by choosing a sample symmetry model. For the complete DSMP analysis and formulation please refer to Kontenis et al. (2016) and Samim et al. (2015). An example of using the DMSP SHG technique is shown in Fig. 12 for wall muscle in *Drosophila* larvae:

#### Interferometric second harmonic generation (I-SHG)

While the coherent nature of SHG advantageously offers additional information about the sample, it is also a weakness since the pattern seen on SHG images results from complex interferences (Rivard et al. 2010; Pinsard et al. 2019c). This can lead to serious imaging artifacts, depending on the microscopic arrangement (Pinsard et al. 2019c), and eventually hide the actual underlying structure (especially in biological samples). Indeed, within the focal volume, dipoles of opposite (respectively same) polarity will destructively (constructively) interfere, leading to areas with a lower (higher) SHG signal. In the image, this results in bright and dark regions without direct correlation with the actual density of harmonophores (compare Fig. 7(e–f)). Hence, to extract quantitative information, it appears necessary to measure the local polarity inside the sample.

It is worth noting that an inversion of polarity (i.e., of the *χ*^(2)^ sign) leads to a π phase shift on the emitted SHG signal (see also bottom row of Fig. 3):

17 $$\begin{array}{c}+{\chi }^{\left(2\right)}\Rightarrow {e}^{i0}{e}^{i\varphi }\Rightarrow {e}^{i\left(\varphi \right)}\\ -{\chi }^{\left(2\right)}\Rightarrow {e}^{i\pi }{e}^{i\varphi }\Rightarrow {e}^{i\left(\varphi +\pi \right)}\end{array}$$

Therefore, the phase of the signal keeps a signature of the polarity within the sample, which can be mapped in each pixel of the image. To do so, the most classical optical technique to record the phase of a signal is based on interferometry. While I-SHG has been originally proposed in 2004 to enable phase measurements on a scanning SHG microscope (Yazdanfar et al. 2004), it was only in 2013 that the technique was first applied to tendon (Rivard et al. 2013a) and later to cartilage (Couture et al. 2015).

**Fig. 13:**
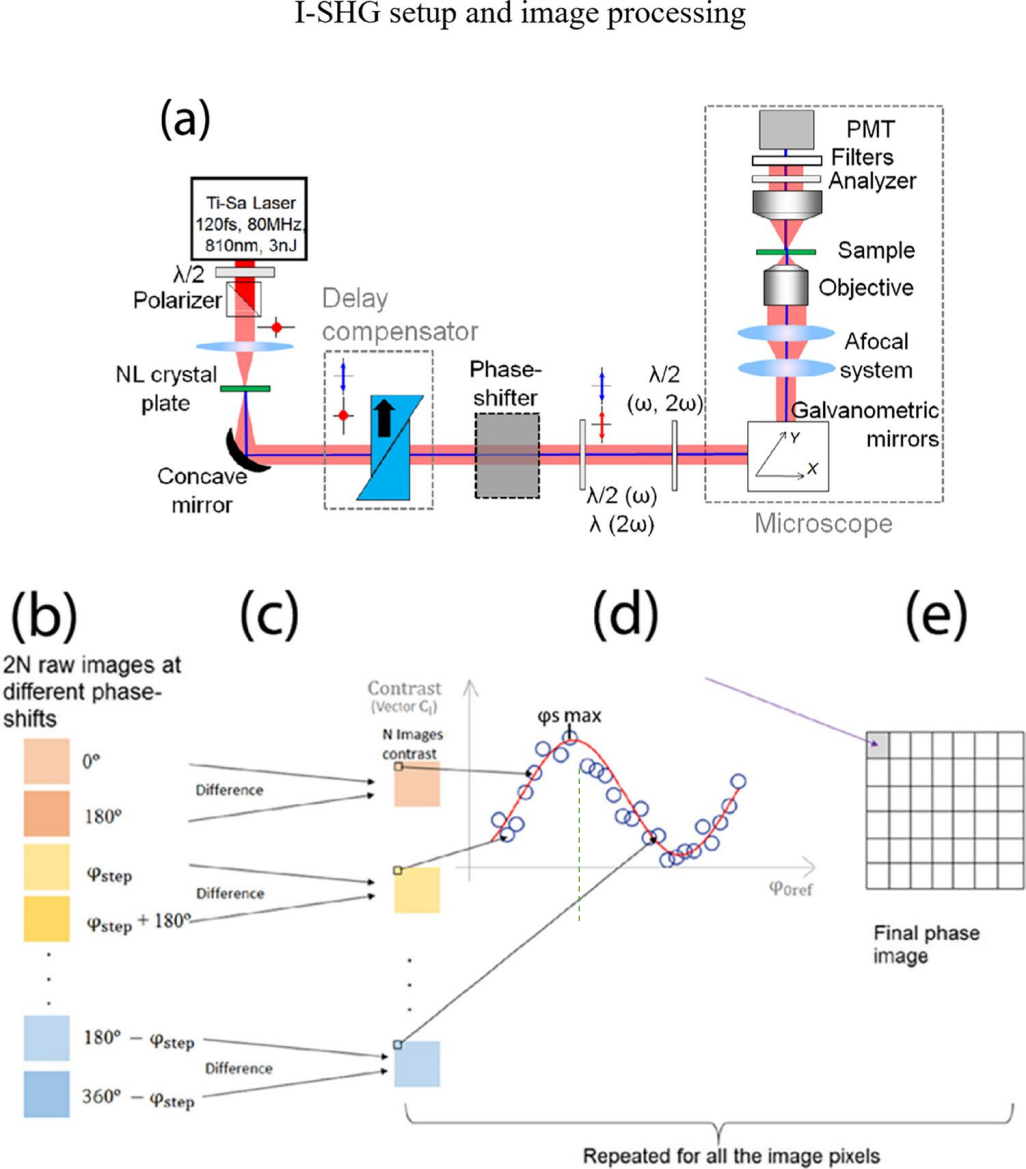
I-SHG principle. (**a**) Example of an I-SHG inverted microscope. The *λ*/2 (half-wave plate) and polarizer are used for power control and afterwards the non-linear crystal plate generates the reference SHG signal. After that, a delay compensator is used to match the optical length of the pump arm and the reference SHG arm superposed along a common path in the interferometer. The polarizations are made parallel after the phase shifter and introduced to the microscope setup for interference between the reference SHG and the sample SHG*.* (**b**, **c**, **d**, **e**) Schematic diagram of the algorithm for calculating the relative I-SHG phase. The 2 N raw images (**b**) are subtracted 2 by 2 to give N contrast images (**c**). In every pixel, the intensity follows a cosine law with respect to the phase shift of the interferogram (**d**), which can be interpolated to find the optical phase and interferometric contrast image (**e**). Extracted from Pinsard (2020)

In this method, the relative polarity of harmonophores is probed by a direct phase measurement. It relies on combining two SHG signals, one from a reference non-linear crystal placed before the microscope (reference SHG) and the second one from the sample (sample SHG), which interfere together (Fig. 13(a)). Since both SHG beams are spatially and temporally coherent, the total intensity on the detector follows the usual two-wave interferometry equation:

18 $${I}_{\mathrm{SHG}}={I}_{\mathrm{s}}+{I}_{\mathrm{ref}}+2{\sqrt{{I}_{\mathrm{s}}{I}_{\mathrm{ref}}}\mathrm{cos}\left({\varphi }_{\mathrm{s}}-{\varphi }_{\mathrm{ref}}\right)}$$

where *I*_*s*_ and *φ*_*s*_ (resp. *I*_ref_ and *φ*_ref_) represent the intensity and the phase of the sample (reference) SHG beam.

Adjusting the phase difference between the two beams enables to record the interferogram and to extract the argument of the cosine (i.e., the relative phase) and its multiplicative factor (the interferometric contrast) by fitting the experimental curve (Fig. 13) (Bancelin et al. 2016). This technique for fitting the cosine from many points is known as phase-shifting interferometry (PSI). In brief, changing the optical path between the reference and the sample arm (Fig. 13(b)) induces a change in the cosine argument from 0 to 2π. To remove the constant term ${I}_{s}+{I}_{\mathrm{ref}}$, two π-phase shifted raw images are subtracted. Then, in every pixel, the experimental cosine (blue circles in Fig. 13(d)) is fitted to determine both the amplitude (interferometric contrast) and the relative phase (${\varphi }_{\mathrm{mat}}$), the interpolated phase of the signal at each point in the material. It is the phase offset found for each interpolated cosine wave at each pixel. The procedure provides phase and interferometric contrast maps.

Various approaches can be used to adjust the phase difference between the two SHG signals, such as a gas cell, variation of distance, a rotating glass plate (Stolle et al. 1996), as well as more advanced approaches, notably the use of an electro-optic phase modulator (EOM) (Pinsard et al. 2019a). Originally, a rotating glass plate was used to induce an optical phase shift between the reference and the sample SHGs (Fig. 13). The refraction at different angles between the SHG and the fundamental and differences in refractive index at these two wavelengths both play a role in changing the relative optical path length between the pump and the SHG when the glass plate is rotated. For a full description of the setup and details on the technique, see Pinsard et al. (2019a) and for a more comprehensive explanation of the phase extraction technique please refer to Pinsard (2020).

**Fig. 14:**
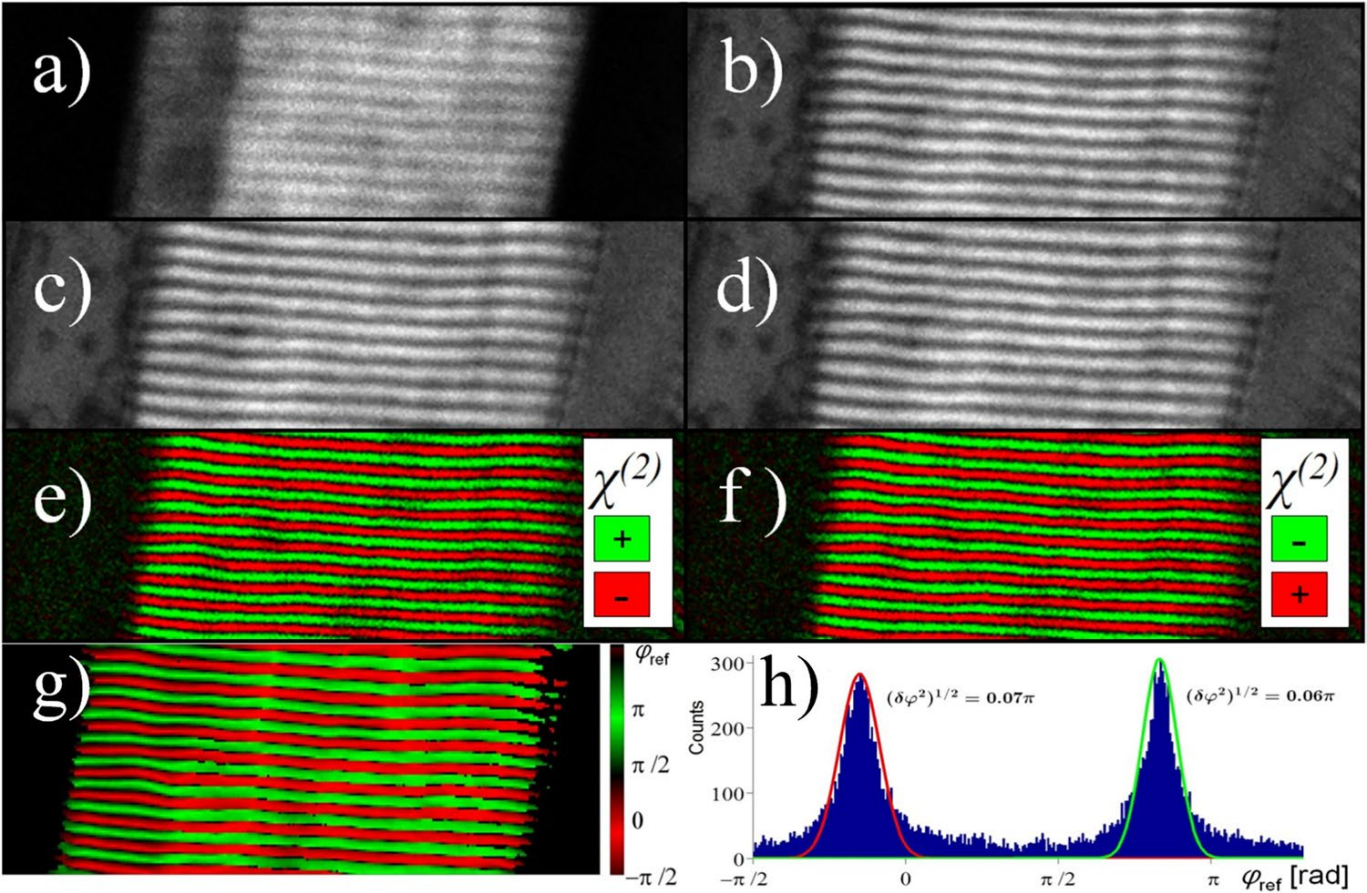
I-SHG imaging in muscle sarcomere adapted from Rivard et al. (2013a). **a**) F-SHG image in the absence of a reference SHG beam. **b**), **c**), **d**) raw I-SHG images acquired with a reference phase of 105°, 285°, and 465° respectively. Panels **e**) and **f**) images resulting from the subtractions of (**c**)—(**b**) and (**d**)—(**c**). Panel **g**) depicts the relative SHG phase in the muscle and (h) the histogram of the relative SHG phase for all pixels in (**g**)

An example of the PSI method can be seen in Fig. 14. In this study, Rivard et al*.* were able to reveal the bipolar structural organization of myosin using I-SHG microscopy (Rivard et al. 2013a). Figure 14(a) displays an SHG image of muscle sarcomeres acquired in the forward direction. The following panels are raw I-SHG images taken with ${\varphi }_{\mathrm{ref}}$ at 105° (b), 285° (c), and 465° (d). Those were the phase shifts resulting in maximal interferometric contrast for this specific image acquisition. Panel (e) and (f) show the results of subtracting two raw I-SHG images taken at ${\varphi }_{\mathrm{ref}}$= 285° and 105° (c–b) and at ${\varphi }_{\mathrm{ref}}$= 465° and 285° (d–c) respectively (Rivard et al. 2013a). The final phase image has been extracted from the 36 images at different reference phase (15° steps) that were taken during this measurement and is shown in panel (g). Lastly, (h) displays the phase histograms associated with image g), highlighting the bimodal distribution of the phase. The distribution of the phase is also represented in histograms to better show some details of the content of that image (h). These results show without ambiguity that, for each sarcomere (white band of the signal) in image (a), there are two associated *χ*^(2)^ domains with opposite polarities.

#### Fast I-SHG microscopy

Because of the optical path difference induced by scanning the laser beam inside the objective of the microscope (and the relay lenses), laser scanning microscopy is not directly applicable to I-SHG. Changing the laser angle onto the objective adds a radial phase distortion in the I-SHG images. I-SHG was thus first developed with a sample-scanning setup (Rivard et al. 2013a), and was later adapted to laser scanning by correcting the phase distortion with a calibration phase map (Bancelin et al. 2016), which improved the imaging time by about 98%, from a few hours down to a few minutes.

However, acquiring an I-SHG image in a few minutes still imposes significant limitations in terms of temporal resolution, since it necessitates that the sample remains steady in the field-of-view along this time frame. Yet, in biological samples, many dynamic processes happen on a shorter time scale: for instance, monitoring cellular mitosis would require a temporal resolution below 30 s to properly resolve moving microtubules (MTs) (Bancelin et al. 2017). Moreover, SHG from MTs is relatively weak, which additionally leads to decreased accuracy of the I-SHG measurements (Pinsard et al. 2019a).

In this context, classical PSI is not optimal since it implies to acquire 18 images of the same zone at different phase shifts (Fig. 13) and leads to long dead time due to the slow speed of the mechanical phase shifter (the glass plate). Therefore, different interferogram points used in the phase extraction (Fig. 13(c)) are separated in time by up to a minute, which leads to significant artifacts due to instabilities.

**Fig. 15:**
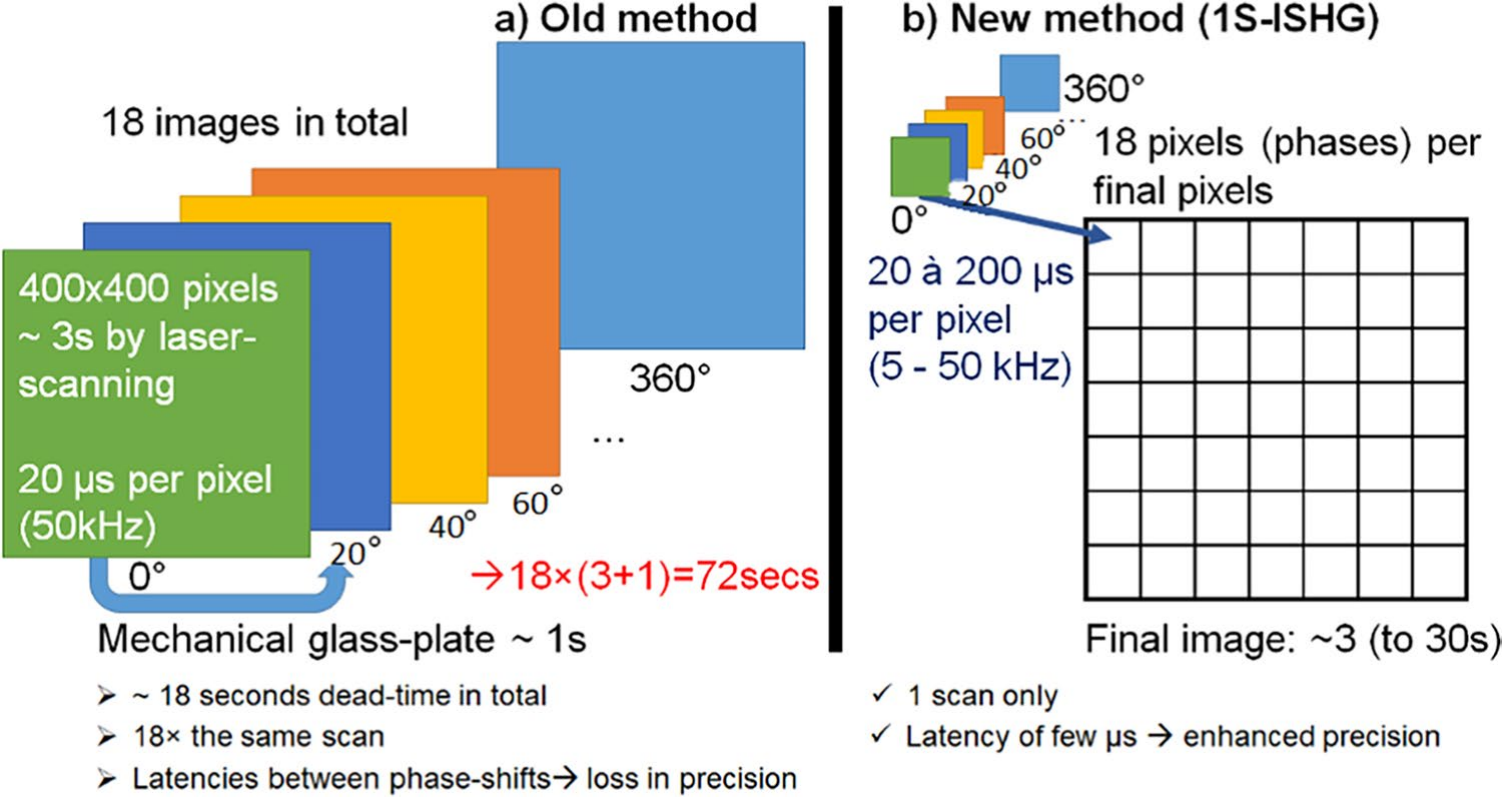
Standard **a**) and fast **b**) methods for phase shift in I-SHG. Extracted from Pinsard (2020)

An improved method, called single-scan I-SHG (1S-I-SHG), has been recently demonstrated and consists in applying the phase shifts within each pixel of the image, rather than between the images (Fig. 15).

**Fig. 16:**
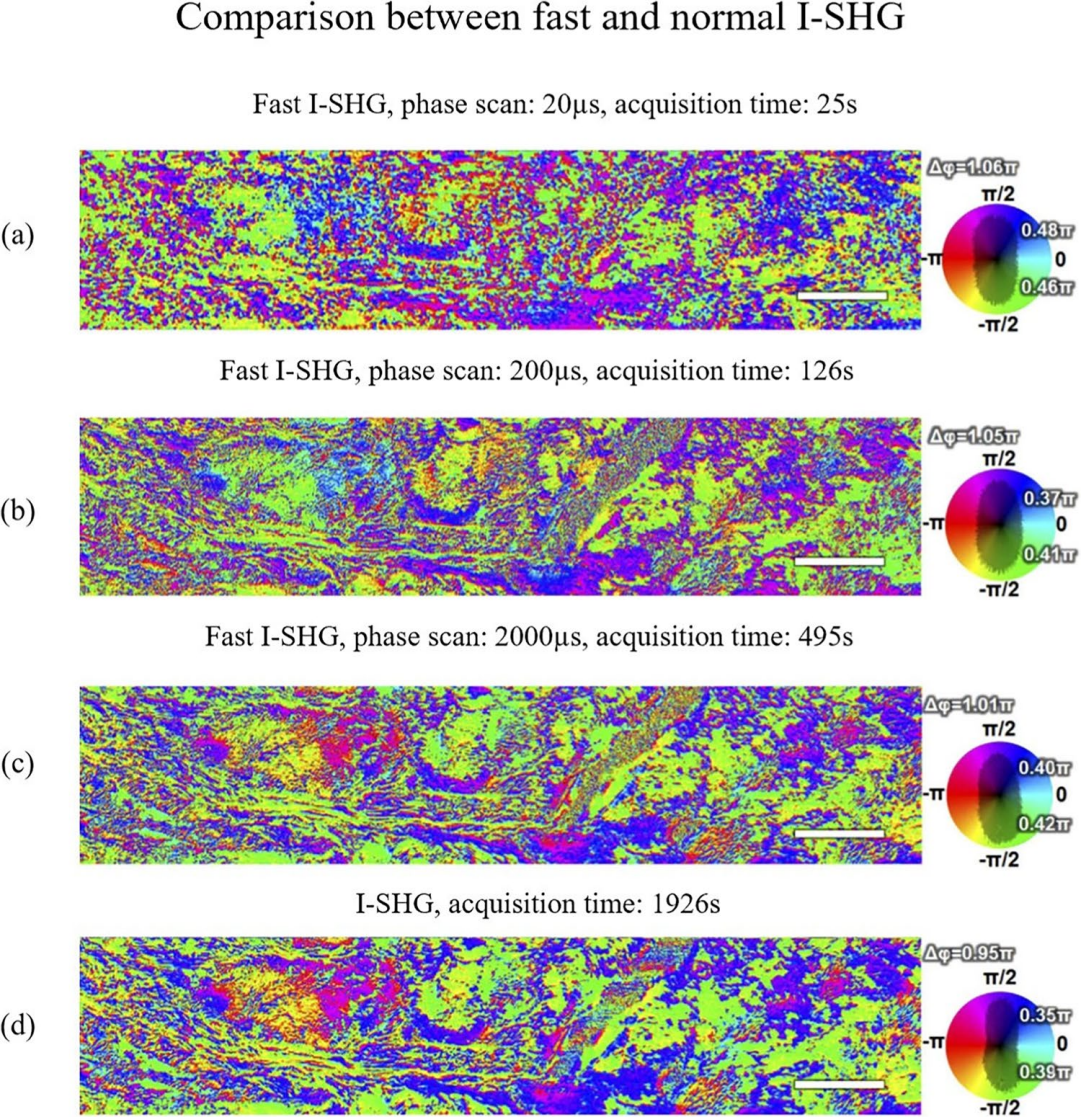
SHG phase-map of an adult horse meniscus with fast I-SHG and normal I-SHG. The scale bar is 50 µm. Panels (**a**), (**b**), (**c**) show fast I-SHG with different phase scan durations and panel (**d**) shows the normal I-SHG at work. (**a**) 20 μs phase scan is acquired in ~ 0.5 min, (**b**) 200 μs phase scan is acquired ~ 2 min, (**c**) 2000 μs phase scan is acquired in ~ 8 min, and (**d**) using the normal I-SHG method, acquisition takes ~ 32 min. Note that reducing the phase scan duration increases the speed of acquisition, but it also increases the phase errors. Nevertheless, even the longest phase scan duration of fast I-SHG (i.e., 2000 μs) cuts the acquisition time by 25% compared to normal I-SHG which is a huge improvement overall. Adapted from Pinsard et al. (2019a)

To that end, the mechanical phase shifter was replaced by an electro-optic modulator (EOM), specifically developed in collaboration with Axis Photonique Inc. (Varennes, Canada), enabling them to tune the phase shift at high speed (up to 50 kHz). This technique results in only one scan of the area, with a settable exposure time (usually between 20 and 200 μs), ensuring only few microseconds of latency between each point of the interferogram (Pinsard et al. 2019a). The amount of time required to image a large area (500 µm × 100 µm) can be seen in Fig. 16 when the fast and normal I-SHG method are used to image the central part of an adult horse meniscus.

Aside from the improved temporal resolution, any sample instability in the implementation would result in image distortion rather than incorrect polarity determination. Consequently, this method appears to be remarkably robust.

#### Wide-field SHG imaging

**Fig. 17:**
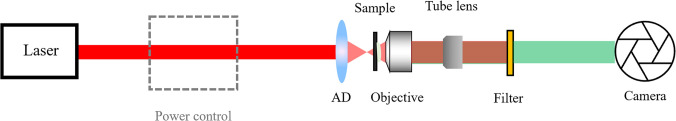
Typical wide-field SHG microscopy setup. The laser light source is in the range of 700–1100 nm. A half-wave plate and a polarizer are used for power control. An achromat doublet lens (AD) is used to focus the incoming laser beam and the sample is placed slightly above the focus to capture a larger FOV. The SHG signal is collected using an objective and a tube lens, spectrally filtered, and detected on a camera. Zhao et al. (2019) Adapted from

Scanning SHG imaging is a well-established method which, over the years, has been successfully used for many applications. However, one of the main implementation limitations is its low imaging throughput (photons detected per frame per second). This drawback impedes its application to label-free imaging of very fast biological processes (millisecond time scale) (So et al. 2013). To overcome this limitation, two strategies can be envisioned: either to increase the scanning speed or to parallelize photon emission. For scanning speed improvement, acousto-optic deflectors (Shao et al. 2012) and resonant scanners (Kirkpatrick et al. 2012) have been successfully used. Yet, they remain ultimately limited by the dwell-time required to generate enough photons to obtain a recordable signal. For the latter strategy, wide-field SHG microscopy appears as the ultimate parallelization, since the complete area of interest is illuminated simultaneously and signals are detected on a pixelated detector (Macias-Romero et al. 2014, 2016). Traditionally, wide-field SHG microscopy was performed using high-energy (µJ) pulses from lasers operated at multi-kHz repetition rate. It has been proven that wide-field SHG microscopy improves imaging throughput by 2–3 orders of magnitude compared to scanning microscopy (Macias-Romero et al. 2014). A typical wide-field SHG setup can be seen in Fig. 17.

**Fig. 18:**
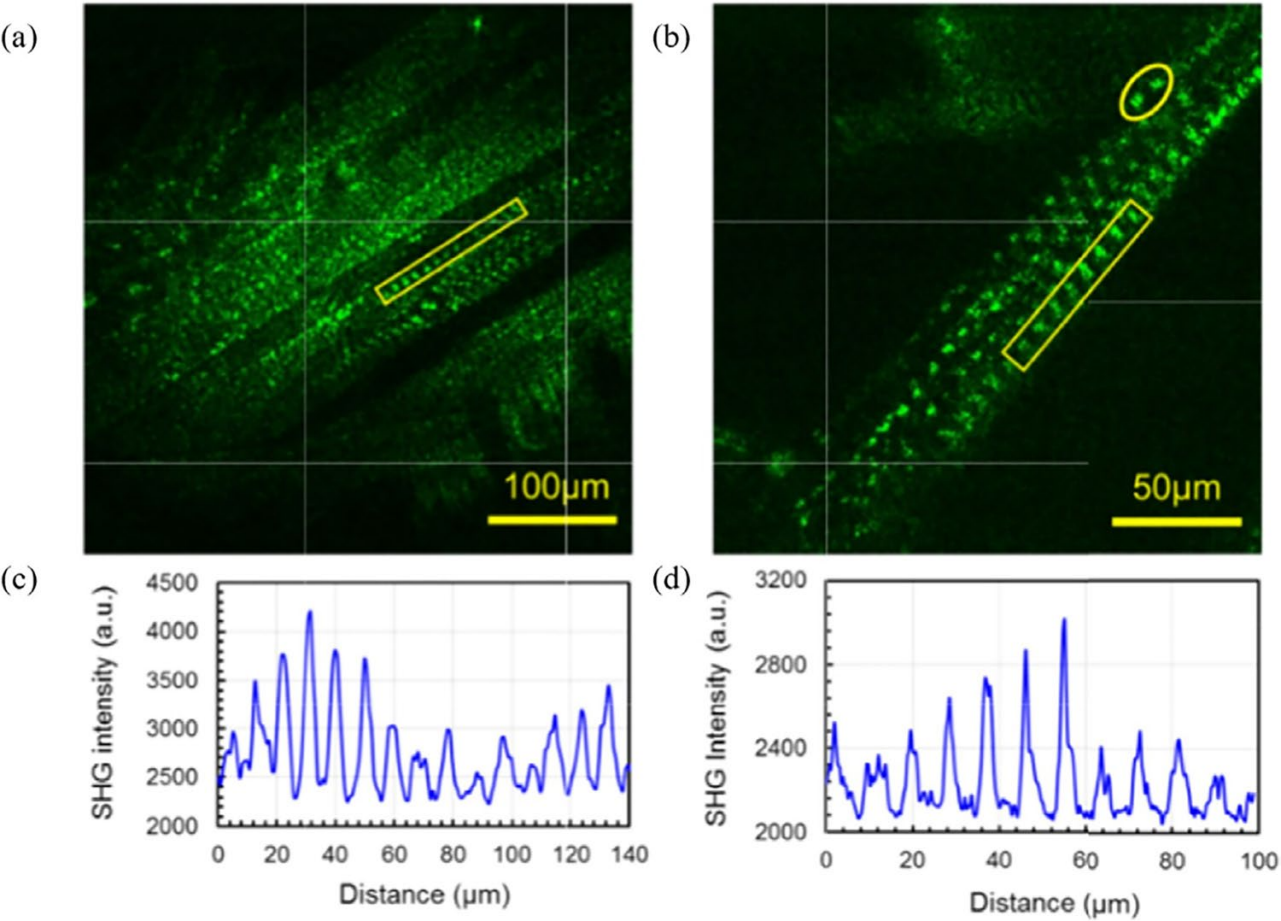
Wide-field SHG images of fixed larval muscle (**a)** 425 µm^2^ and (**b**) 213 µm.^2^ area with a frame integration time of 100 ms. Panels (**c**) and (**d**) represent the SHG intensity profiles of (**a**) and (**b**) respectively from the designated regions of interests in the images. This method provides single shot imaging of large areas and is used to acquire live larvae contractions. Extracted from Zhao et al. (2019)

Due to the delicacy of living cell samples, particular care must be taken to avoid photodamage. Several studies investigated light damage in wide-field SHG microscopy for different cell lines allowing to determine a range where pulse energy, and hence heat deposition remains below the damage threshold of the samples (Zhao et al. 2019). In recent advances, a high repetition rate (MHz) wide-field SHG microscope has been designed for live imaging of contracting muscle tissue that utilizes laser pulses with pulse energy as low as approximately 60 nJ per pulse (Fig. 18).

Lastly, holographic SHG microscopy, a variant of wide-field SHG, has been proposed to make use of the signal phase (Shaffer et al. 2010a, b). Other methods beside wide-field SHG microscopy also exist for improving the image acquisition speed, and we suggest (Wu et al. 2021) for a recent comprehensive review of these methods.

### SHG and enhanced SHG in neurons

#### The nervous system and neuron structure

**Fig. 19:**
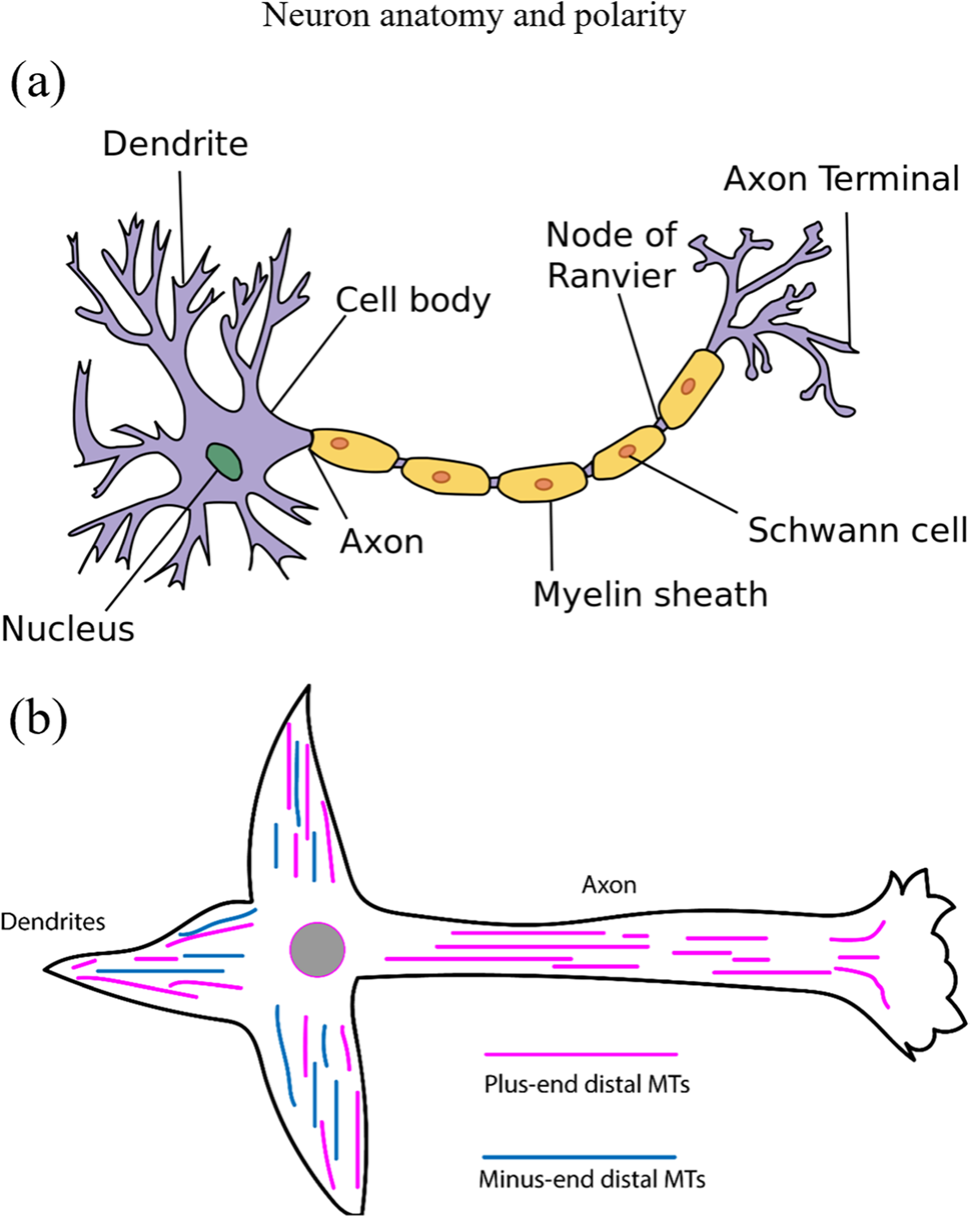
(**a**) Anatomy of a neuron from soma to the synapse. Extracted from Neuron description (2019). (**b**) Neuron polarity diagram. Adapted with permission from Baas and Lin (2011)

The nervous system is a *sine qua non* organ for most living animals, responsible for information processing and transmission (Stufflebeam 2006). As depicted in Fig. 19, neurons have a cell body called the soma, which contains the nucleus of the neuron. The receiving branches of the neuron are called dendrites, where most of the incoming signals are integrated (Fowler et al. 2013). The outgoing signal drives through a structure called the axon. Although a neuron can have many dendrites, it will always have only one axon. At the end of the axon, there are the axon terminals and synapses that contain the neurotransmitters necessary for chemical communication between the neurons.

Studying neurons and their building components is one of the highest challenges of our times since many of their mechanisms and dysfunctions remain unknown. The following section will cover how well-suited SHG microscopy is to study these structures.

#### SHG microscopy in neurons

Traditionally, neuronal mechanisms have been studied using electrophysiology in which electrodes are inserted into the neurons to measure the electrical potentials and currents. This approach is considered the gold standard to study neuronal activity and has led to invaluable information about neuron functioning. Nevertheless, this approach still presents several significant limitations. Firstly, it requires a rather invasive protocol and remains challenging to use in living animals (Zhang et al. 2006). Moreover, despite recent advances, patch clamping is bounded to record data from a limited number of neurons, drastically impeding the investigation of a neuronal network (Stuart and Palmer 2006).

**Fig. 20:**
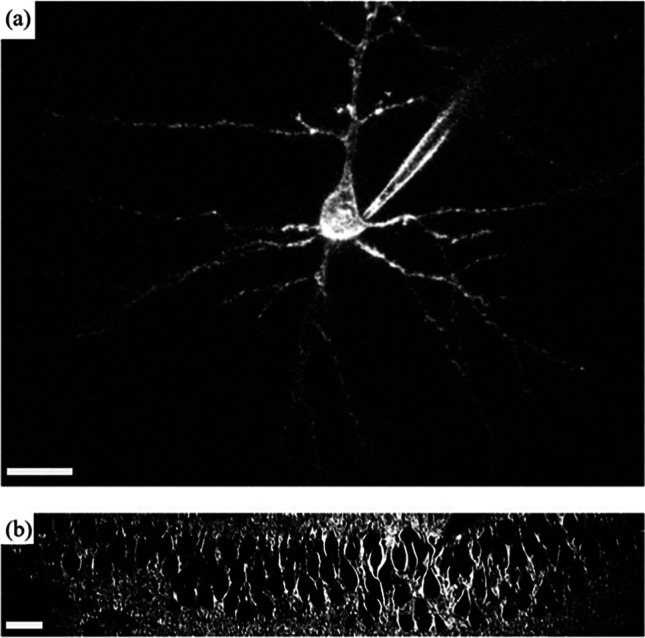
Single neuron and neuron population using FM-4–64 dye and SHG imaging. Scale bars: 20 µm. Panel (**a**) shows a single pyramidal neuron from a mouse visual cortex that has been injected with the dye and imaged using SHG microscopy. Panel (**b)** shows a multitude of pyramidal neurons bathed and labeled by a SHG chromophore and imaged using SHG microscopy. Extracted with permission from Jiang and Yuste (2008)

To overcome such shortcomings, optical methods appear highly desirable by offering the necessary flexibility to complement such electrophysiological measurements. 2PEF and SHG microscopy have found many applications in neuroscience and are vastly gaining popularity because they provide complementary access to distinct features. Although most SHG microscopy experiments have been based on endogenous cell properties, SHG dyes have also been used in different studies (Campagnola et al. 2001; Nemet et al. 2004; Dombeck et al. 2005; Nuriya et al. 2006; Jiang et al. 2007). Using FM 4–64 dye, Dombeck et al. demonstrated a huge improvement in signal-to-noise ratio (SNR) over fluorescent probes (Dombeck et al. 2005). Using the same dye, Nuriya et al. were able to demonstrate for the first time that action potentials enter dendritic spines (Nuriya et al. 2006), and later characterized the SHG response to an action potential and its propagation from the soma to the axons (Nuriya and Yasui 2010). In parallel, Nemet et al. reported that all trans retinal chromophores are suitable candidates for SHG neuronal membrane imaging (Nemet et al. 2004). Jiang et al. showed that the limited SNR obtained in the previous studies could be overcome (Fig. 20) using photon counting detection (Jiang and Yuste 2008) and later reported that the potential sensing capacity of FM 4–64 originates from electro-optical mechanisms (Jiang et al. 2007).

To summarize, among many existing tools and methods for neuronal mechanism studies, optical imaging techniques can be advantageous for studying various aspects of the neurons. SHG microscopy is reliant on the cell properties for SHG signal production. In some studies, SHG dyes are necessary to further improve the SNR and the contrast in the images (Nemet et al. 2004; Dombeck et al. 2005; Jiang et al. 2007; Nuriya and Yasui 2010).

#### SHG microscopy in microtubules

In neuroscience, one of the interesting structures that has been investigated using SHG microscopy are microtubules (MTs). MTs are among the most important cytoskeleton filaments and their functionality encompasses the maintenance of the cell integrity and the morphology or regulation of intracellular trafficking, while also playing an important role in cell division (Baas et al. 1988; Baas and Lin 2011; Alushin et al. 2014; Kapitein and Hoogenraad 2015). MTs are structured as hollow tubes with a 25-nm outer diameter that is made of two dimers *α*- and *β*-tubulin. When these two heterodimers bind in a head–tail manner, they create a linear protofilament polymer (Horio and Murata 2014; Kapitein and Hoogenraad 2015). MTs are fundamentally polar because all protofilaments are parallel to each other and all the dimers comprising the filament share the same orientation (Horio and Murata 2014).

Many studies have focused on how MTs produce SHG (Dombeck et al. 2003; Kwan et al. 2008; Kwan 2013; van Steenbergen et al. 2019). One of the recent highlights (van Steenbergen et al. 2019) reports that the number, organization, and polarization all play an important role in the formation of SHG signal from MT (van Steenbergen et al. 2019).

Although the polarity was not the focus of these studies, the MT polarity was later deduced, using the protein plus method which tags the microtubule-associated protein (MAP) with a fluorophore and the tagged MAP then binds to the positive end of MTs (Akhmanova and Hoogenraad 2005; Baas and Lin 2011). Combined with SHG microscopy, it was shown that the MTs polarity in the axons is well-defined and always the same, with the minus end pointing to the cell body and the positive end pointing to the axon terminals, where neurotransmission takes place (Baas and Lin 2011). However, this method is invasive since it uses fluorescent markers for determining the polarity of the MTs. Importantly, while paraformaldehyde is the gold standard in cell fixation, this fixation method was also investigated in this study and it causes drastic losses of SHG signal which reveals that it changes the protein conformation (van Steenbergen et al. 2019). In contrast, MTs polarity in dendrites remains poorly characterized, but seems to be not so well-defined (Baas et al. 1988). Even if some previous studies have suggested that there might be a mix of polarity in dendrites (Dombeck et al. 2003) and that domains of polarity exist among them (Kwan et al. 2008), these claims remain hypothetical and a full characterization is required to verify them. Notably, many questions remain unanswered, e.g., why do axons have uniform polarity, but dendrites do not? Is this mixed polarity functionally relevant? SHG and specifically I-SHG are great candidates for studying the underlying mechanisms of the dendrites and the relevance of their polarity in their operation.

**Fig. 21:**
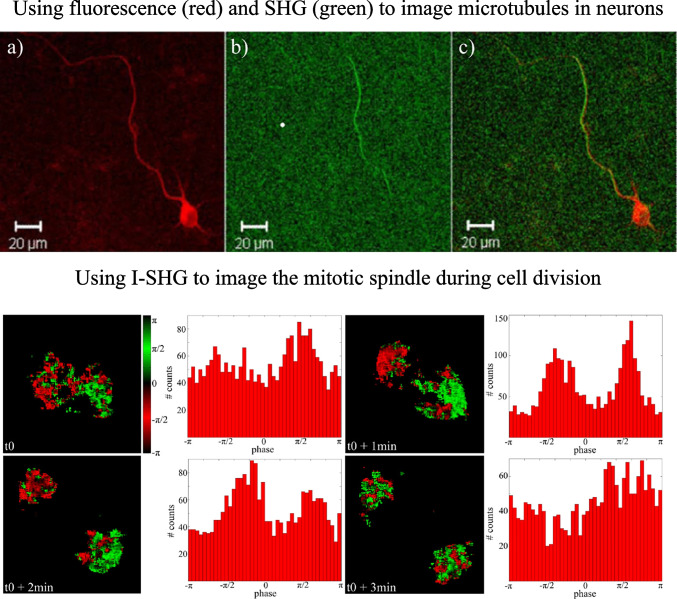
Microtubule imaging in neuron (top) and mitotic spindle (bottom). Complementation of neuron imaging using fluorescence and SHG (top). In panel **a**), only TauRFP (tau red fluorescent protein) dye is visible in the image of the neuron. In panel **b**), we only see the SHG image of the neuron. Finally, **c**) is a combination of the fluorescence and the SHG images to benefit from both imaging techniques (Stoothoff et al. 2008). Image and histogram of SHG phase in the mitotic spindles (bottom). The red and green pixels are π-phase shifted signals. At the beginning of the metaphase (t0), the two poles are starting to have opposite polarities. At the end of the metaphase (t0 + 1 min) and the beginning of the anaphase (t0 + 2 min), a more uniform polarity can be seen where one pole is red and the other pole is green. At the end of the anaphase (t0 + 3 min), a mix of red and green pixels can be seen in both poles which means that the two poles have a random polarity. Reproduced under CC BY 4.0 from Bancelin et al. (2017)

Lastly, embryogenesis is an entire field in developmental biology, in which SHG microscopy has been instrumental in providing time-lapse images of the distinct stages of cell division. Specifically, SHG rises and falls have been used to investigate the dynamics of mitotic spindles, composed of highly oriented MTs, in different embryos, including *Caenorhabditis elegans*, zebra fish, mouse, rat, and sea urchins (Kwan 2013). While several methods allow to study the polarity of MTs, most of them are invasive and I-SHG microscopy appears to be a promising non-invasive alternative. In a study using the I-SHG technique, Bancelin et al. (Bancelin et al. 2017) successfully mapped the polarity of MTs forming the mitotic spindle during cell division in zebrafish embryos as shown in the bottom part of Fig. 21. While the polarity of MTs in mitotic spindles had been previously studied indirectly with a combination of SHG and fluorescence microscopy (Yu et al. 2014), this was the first direct evidence of change in MTs polarity upon mitosis. This achievement was made possible by the advances in the I-SHG imaging speed, as discussed in “Interferometric second harmonic generation (I-SHG).” It was found that at different stages of the cell division, the SHG signal varied due to the change of alignment and polarity of the MTs’ network. Bancelin et al. observed the SHG signal during various phases. First occurring in the pro-metaphase, the signal further increased in the metaphase and anaphase, and gradually vanished during the telophase when the mitotic spindle uncondensed. Besides the SHG intensity, they could extract the polarity of MTs during each phase. They observed that at the beginning of the metaphase and the end of anaphase, MTs had a mixed polarity revealing a more disorganized structure. In contrast, at the end of the metaphase and the beginning of the anaphase, the MTs are highly aligned with uniform polarity (Bancelin et al. 2017). This study showcased the power of the I-SHG microscopy technique and how it would be advantageous to use this method for studying the polarity in dendrites and other neuronal activities. More generally, SHG and advanced SHG microscopy are versatile tools that were utilized in many MTs studies. They have shown promising potential and are a great candidate for in-depth studies of different aspects and unknown mechanisms of MTs and related diseases (Stoothoff et al. 2008; Bancelin et al. 2017; van Steenbergen et al. 2019).

## Conclusion and prospects

Over the past two decades, SHG microscopy has become an invaluable tool in bio-imaging and neuroimaging. Many studies illustrate its potential to investigate non-centrosymmetric biological structures such as fibrillar collagenous tissues (Chen et al. 2012), tendon (Freund and Deutsch 1986; Légaré et al. 2007; Hase et al. 2016), cartilage (Yeh et al. 2005), cornea (Han et al. 2005; Latour et al. 2012), sclera (Han et al. 2005), fascia (Rivard et al. 2010), meniscus (Pinsard et al. 2019b), muscle (Mohler et al. 2003; Ralston et al. 2008; Wallace et al. 2008; Rivard et al. 2013a, b, 2014), MTs (Dombeck et al. 2003, 2005; Kwan et al. 2008; Kwan 2013; Bancelin et al. 2017), otoconia (Brittain et al. 2022), the origin of SHG signal in neurons (Dombeck et al. 2003; van Steenbergen et al. 2019), and how it can a be great tool for tauopathies (Stoothoff et al. 2008) and tubilinopathies (Alata et al. 2022). While originally limited to point-scanning imaging of endogenous structures, over the years, many groups have demonstrated innovative approaches to minimize the invasiveness and to improve the imaging throughput, notably through wide-field imaging or the development of specific SHG probes, constantly pushing the frontier of SHG imaging into new systems and structures.

In this context, conceptual and technological advances in SHG microscopy continue to define a fast-progressing frontier in biophotonics. Aiming to improve the spatial resolution by means of coherent structured illumination (Nikolenko et al. 2008) and utilizing post-processing methods such as pixel reassignment (Wang et al. 2021; Raanan et al. 2022), increasing the imaging depth through adaptive optics approaches (Hoover and Squier 2013) or pushing non-linear imaging into the spectroscopic realm using hyperspectral microscopy approaches based on sum-frequency generation (Hanninen et al. 2017) are all examples of this fast ongoing progress.

Despite many advances, a fully quantitative interpretation of SHG images remains elusive owing to the coherent nature of the process involved. While the different approaches presented in this review, notably F/B-SHG, P-SHG, I-SHG, CD-SHG, and Stokes vector–based SHG all appear as relevant pieces to this puzzle, their combination in the same instrument has yet to be done but could potentially provide a definitive answer to this long-lasting topic.

With its tremendous advantages, SHG microscopy still requires overly expensive equipment and specialized training, which impedes its larger use in routine biomedical practice. This is particularly evident for the more advanced SHG techniques that rely on state-of-the-art optical implementation and complex hardware system. The recent advancement in laser technology has led many groups to shift away from the gold standard of Ti:sapphire lasers towards more robust and power-efficient fiber and semiconductor lasers enabling smaller and more efficient SHG microscopes (James and Campagnola 2021). This crucial simplification and cost reduction is expected to open new perspectives for biomedical applications of SHG microscopy. Such wide application would be promoted by the progress in endoscopic SHG, which has gained popularity in recent years (Kučikas et al. 2021). There are still significant technological challenges that need to be overcome to make this technology more accessible, but the efforts required to solve these technological challenges would be matched with even greater potential reward, like enabling in vivo imaging of organs.

Besides hardware implementation, software analysis and computational approaches for enhancing imaging capabilities have also made great strides in microscopy. These computational advancements complement the optical setups and even correct some of their flaws and shortcomings in imaging. Notably, fast image processing has been made possible in recent years thanks to improvements in graphical processing units and field-programmable gate arrays that can process large amounts of raw data at high speed. In addition, machine learning is currently revolutionizing many fields including image processing and has naturally made its way into SHG microscopy. For example, a few groups recently ventured to develop deep learning algorithm based on neuronal networks to classify and diagnose cancer using SHG footprints (Huttunen et al. 2018; Kistenev et al. 2019; Mirsanaye et al. 2021; Preston et al. 2022).

Lastly, many current imaging systems are unique setups, customized differently in each lab (Wu et al. 2021). A unification and standardization of the imaging process appears highly desirable for reproducibility and portability.

Regardless of the challenges and limitations we mentioned, SHG and non-linear optical microscopy imaging modalities provide a plethora of information that is not readily available with traditional linear or incoherent optical imaging techniques. With all the technological advancements in optics, machine learning, and laser technology, non-linear imaging modalities are only going to get better and much simpler over time, opening new horizon for widespread applications in both fundamental science and medical applications (James and Campagnola 2021).

## Data Availability

Review paper, Not Applicable.

## Code availability

Review paper, Not Applicable.
